# Elevated CO_2_ alters soybean physiology and defense responses, and has disparate effects on susceptibility to diverse microbial pathogens

**DOI:** 10.1111/nph.20364

**Published:** 2025-01-09

**Authors:** Melissa Bredow, Ekkachai Khwanbua, Aline Sartor Chicowski, Yunhui Qi, Matthew W. Breitzman, Katerina L. Holan, Peng Liu, Michelle A. Graham, Steven A. Whitham

**Affiliations:** ^1^ Department of Plant Pathology, Entomology & Microbiology Iowa State University Ames 50011 IA USA; ^2^ Department of Statistics Iowa State University Ames 50011 IA USA; ^3^ W. M. Keck Metabolomics Research Laboratory Iowa State University Ames 50011 IA USA; ^4^ United States Department of Agriculture (USDA), Agricultural Research Service (ARS), Corn Insects and Crop Genetics Research Unit and Department of Agronomy Iowa State University Ames 50011 IA USA

**Keywords:** bean pod mottle virus, carbon dioxide, *Fusarium virguliforme*, *Glycine max*, plant immunity, *Pseudomonas syringae*, *Pythium sylvaticum*, soybean mosaic virus

## Abstract

- Increasing atmospheric CO_2_ levels have a variety of effects that can influence plant responses to microbial pathogens. However, these responses are varied, and it is challenging to predict how elevated CO_2_ (*e*CO_2_) will affect a particular plant–pathogen interaction. We investigated how *e*CO_2_ may influence disease development and responses to diverse pathogens in the major oilseed crop, soybean.
- Soybean plants grown in ambient CO_2_ (*a*CO_2_, 419 parts per million (ppm)) or in *e*CO_2_ (550 ppm) were challenged with bacterial, viral, fungal, and oomycete pathogens. Disease severity, pathogen growth, gene expression, and molecular plant defense responses were quantified.
- In *e*CO_2_, plants were less susceptible to *Pseudomonas syringae* pv. *glycinea* (*Psg*) but more susceptible to bean pod mottle virus, soybean mosaic virus, and *Fusarium virguliforme*. Susceptibility to *Pythium sylvaticum* was unchanged, although a greater loss in biomass occurred in *e*CO_2_. Reduced susceptibility to *Psg* was associated with enhanced defense responses. Increased susceptibility to the viruses was associated with reduced expression of antiviral defenses.
- This work provides a foundation for understanding how future *e*CO_2_ levels may impact molecular responses to pathogen challenges in soybean and demonstrates that microbes infecting both shoots and roots are of potential concern in future climatic conditions.

## Abstract 2

See also the Commentary on this article by Sanchez‐Lucas & Luna, **246**: 2380–2383.

## Introduction

Atmospheric carbon dioxide (CO_2_) levels are steadily rising and by some estimates, are predicted to increase to 550 parts per million (ppm) or more by mid‐century. Increasing CO_2_ levels, in combination with more extreme and unpredictable weather events, are expected to have significant impacts on global food security. The majority of plants on Earth use C_3_ photosynthesis, with relative atmospheric [O_2_] and [CO_2_] favoring photorespiration or photosynthesis, respectively (Pinto *et al*., 2014). As a result, elevated CO_2_ (*e*CO_2_) stimulates photosynthesis, generally increasing plant biomass and yield, providing a potential benefit to crop production (Dusenge *et al*., 2019; Ainsworth & Long, 2021). However, this CO_2_ ‘fertilization effect’ depends on a number of factors, including water and nutrient availability and optimal growth temperatures (Ainsworth & Long, 2021). Moreover, suppression of photorespiration, once thought to be a wasteful process, has been associated with poor growth (Timm & Bauwe, 2013), lower nutrient and protein content (Taub *et al*., 2008; Broberg *et al*., 2017), and reduced abiotic stress tolerance (Voss *et al*., 2013) under ambient atmospheric conditions (*a*CO_2_).

Another possible impact of *e*CO_2_ is changes in the occurrence and severity of plant diseases. Pests and diseases that affect plant quality and yield pose one of the greatest challenges to crop production and are responsible for 17–30% of total yield losses of major crops (Savary *et al*., 2019). Climate change is expected to cause latitudinal shifts in disease pressure associated with the migration of important plant pathogens to new geographic regions (Bebber *et al*., 2013; Chakraborty, 2013; Raza and Bebber, 2022) as well as changes in pathogen virulence or aggressiveness (Pangga *et al*., 2007; Lake & Wade, 2009; Aguilar *et al*., 2015; Singh *et al*., 2023). Additionally, *e*CO_2_ has been demonstrated to alter interactions between plants and their attackers through changes in plant architecture, physiology, and molecular defense responses (Velásquez *et al*., 2018; Bazinet *et al*., 2022).

Plant disease development relies on three factors: a susceptible plant host, a virulent pathogen, and environmental conditions conducive to infection (Stevens, 1960; Grulke, 2011). Over the past decade, tremendous advances have been made in our understanding of plant–microbe interactions (Ngou *et al*., 2022; Petre *et al*., 2022); however, it is still not well‐understood how changes in environmental conditions shape these interactions. Several mechanisms by which *e*CO_2_ affects defense responses have been reported, including changes in leaf nutrition (Ryalls *et al*., 2015; Sun *et al*., 2016), stomatal density (Li *et al*., 2015; Zhou *et al*., 2017), host metabolism (Matros *et al*., 2006; Ode *et al*., 2014), and redox homeostasis (Mhamdi & Noctor, 2016; Noctor & Mhamdi, 2017; Foyer & Noctor, 2020; Ahammed & Li, 2022). Altered levels of defense‐related phytohormones, salicylic acid (SA), jasmonic acid (JA), and ethylene (ET), have also been reported (Zhang *et al*., 2015; Pan *et al*., 2019; Zhou *et al*., 2019). In general, constitutive and/or pathogen‐induced SA levels have been reported to be higher in the foliar tissues of C_3_ plants grown under *e*CO_2_ (Bazinet *et al*., 2022), suggesting that plants could be more resistant to viral and biotrophic pathogens (Vlot *et al*., 2009; Murphy *et al*., 2020). However, the extent and directionality of these responses are highly variable between studies (Bazinet *et al*., 2022), which may reflect species, cultivar, or ecotype‐specific CO_2_ adaptations, as well as differences in additional environmental growth conditions. For example, in tomato (*Solanum lycopersicum*), *e*CO_2_ increased SA biosynthesis, and plants displayed higher resistance to leaf curl virus, tobacco mosaic virus, and the hemibiotrophic bacterial pathogen *Pseudomonas syringae* pv. *tomato* (*Pst*DC3000), but were more susceptible to the necrotrophic fungal pathogen *Botrytis cinerea* (Huang *et al*., 2012; Zhang *et al*., 2015). By contrast, *e*CO_2_ increased JA‐dependent responses in Arabidopsis (*Arabidopsis thaliana*), enhancing resistance to *B. cinerea* and reducing resistance to *Pst*DC3000 (Zhou *et al*., 2019). These discrepancies highlight the need for direct investigations of pathosystems involving important crop species rather than relying on generalized information from model systems.

Soybean (*Glycine max* (L.) Merr.) is the most widely grown protein and oilseed crop, accounting for *c*. 329 million tons of global crop production in 2022 (http://www.worldagriculturalproduction.com/). The effect of *e*CO_2_ on soybean physiology has been investigated at various scales (Ainsworth & Long, 2021; Li *et al*., 2021). In general, soybean yield potential and root nodule mass increase under *e*CO_2_ (Dermody *et al*., 2008) with no significant impact on plant nutrient or protein content (Myers *et al*., 2014). *e*CO_2_ has also been associated with changes in leaf endophyte communities (Christian *et al*., 2021; Gonçalves *et al*., 2021), rhizospheric bacterial communities (Yu *et al*., 2016), and responses to herbivore attack (Casteel *et al*., 2008; O'Neill *et al*., 2010; Paulo *et al*., 2020). However, only one study has reported the effect of predicted future CO_2_ atmospheric conditions (550 ppm) on soybean diseases (downy mildew, brown spot, and sudden death syndrome (SDS)) by monitoring the incidence and severity of some naturally occurring diseases at the Soybean Free Air Concentration Enrichment (SoyFACE) facility (Eastburn *et al*., 2010). As soybean is susceptible to numerous economically important pathogens, including bacteria, fungi, oomycetes, viruses, and nematodes that are expected to be affected by climate change (Whitham *et al*., 2016; Roth *et al*., 2020), a formal investigation of how *e*CO_2_ impacts susceptibility to diverse pathogens is needed. Moreover, the effects of *e*CO_2_ on molecular defense responses has not yet been investigated in this species.

In this study, we assessed the effects of *e*CO_2_ on soybean defense response in plants grown under the current [*a*CO_2_] of 419 ppm vs an [*e*CO_2_] of 550 ppm. Using the soybean–*P. syringae* pathosystem, we assessed innate immune responses and disease susceptibility and conducted transcriptomic analyses to gain a global understanding of how *e*CO_2_ levels affect interactions in this pathosystem. We also conducted infection experiments using two foliar viral pathogens, bean pod mottle virus (BPMV) and soybean mosaic virus (SMV), and two filamentous root pathogens, *Fusarium virguliforme* and *Pythium sylvaticum*, and demonstrate that *e*CO_2_ exerts differential effects on interactions with these diverse microbial pathogens. Our work provides a foundation for future studies investigating the molecular interplay that regulates defense responses in *e*CO_2_ and identifies pathogens of potential concern in predicted future atmospheric conditions.

## Materials and Methods

### Plant growth and maintenance

Soybean (*Glycine max* (L.) Merr. cv Williams 82) plants were grown in chambers at the Iowa State University Enviratron facility (Bao *et al*., 2019) with lighting, humidity, and temperature conditions as illustrated in Supporting Information Fig. S1. [CO_2_] maintained at 419 ppm represented *a*CO_2_ and 550 ppm represented future atmospheric conditions (*e*CO_2_) (Jaggard *et al*., 2010). For each condition, three replicate growth chambers were used to provide biological replicates. Plants were grown in LC‐1 potting soil mix (Sungro, Agawam, MA, USA) and fertilized weekly with 15‐5‐15 Cal‐Mag Special (G99140; Peters Excel, St. Louis, MO, USA).

### Physiological measurements

Shoot biomass, photosystem II (PSII) activity (quantum yield of fluorescence; ΦPSII activity), and stomatal conductance (gas exchange rate; *g*_sw_) were measured for 15 plants per CO_2_ treatment. ΦPSII and *g*_sw_ were obtained using a LI‐600 Portable System (LI‐COR Biosciences, Lincoln, NE, USA) during the VC‐V4 growth stages (Kumudini, 2010). ΦPSII measurements were conducted on the adaxial surface of unifoliate leaves of 14‐d‐old plants and the newest fully expanded trifoliolate leaves of 3‐ and 5‐wk‐old plants. *g*_sw_ measurements were simultaneously collected from the abaxial surface. At 21 d after planting (dap), leaves were collected from eight plants per treatment and chamber, yielding three biological replicates per treatment for QuantSeq 3′ mRNA‐Seq analyses (48 samples for QuantSeq dataset 1). Samples were collected between 09:00 h and 10:00 h for all replicates. At 35 dap, shoots were collected to determine fresh weight and dry weight.

### Stomatal density, aperture, and index measurements

Stomatal density and aperture measurements were assessed from 10 leaf samples, while stomatal index measurements were taken from five leaf samples for each CO_2_ treatment at 21 dap. Impressions were made from the abaxial leaf surfaces using clear nail varnish (Ceulemans *et al*., 1995). Stomata were imaged using light microscopy, and counted in three randomly selected fields of view per leaf sample. Stomatal aperture and stomatal index measurements were conducted as described (Sultana *et al*., 2021; Zhu *et al*., 2021).

### Reverse transcription quantitative polymerase chain reaction and quantitative polymerase chain reaction analyses

Total RNA was isolated from *c*. 50 mg of leaf tissue using TRIzol Reagent (Thermo Fisher Scientific, Waltham, MA, USA). Subsequently, 2 μg of RNA was reverse transcribed using the Maxima First Strand cDNA Synthesis Kit (Thermo Fisher Scientific). Reverse transcription quantitative PCR (RT‐qPCR) was conducted using 2× PrimeTime Gene Expression Master Mix (Integrated DNA Technologies, Coralville, IA, USA) and multiplexed probes designed for *Pathogenesis‐Related Protein 1* (*PR1*) (Glyma.13G251600), *Kunitz Trypsin Inhibitor 1* (*KTI1*) (Glyma.08G342000), BPMV, or SMV (Table S1). Expression of *Dicer‐Like 2* (*DCL2*) (Glyma.09 g025300) and *Argonaute 1* (*AGO1*) (Glyma.09 g167100) was assessed using iTaq Universal SYBR Green Supermix (Bio‐Rad). *F. virguliforme* and *P. sylvaticum* were quantified in soybean roots at 14 and 35 dap, respectively. DNA was extracted using the CTAB method (Rogers & Bendich, 1994), and qPCR was performed using primers specific to each pathogen (Table S1). *S‐phase kinase‐associated protein 1* (*Skp1*) (Glyma.08G211200) was used as an internal reference for all experiments (Beyer *et al*., 2021). Primers and probes were designed from cultivar Williams 82 reference sequence (version Wm82.a4.v1, Schmutz *et al*., 2010) using PrimerQuest Tool (Integrated DNA Technologies).

### Immune signaling assays

Oxidative species production was monitored in 14‐d‐old soybean plants (Bredow *et al*., 2019) using leaf disks collected from 16 plants per CO_2_ treatment. The elicitor solution contained 100 μM luminol (Sigma‐Aldrich), 10 μg ml^−1^ horseradish peroxidase (Sigma‐Aldrich), with or without 100 nM flg22 (VWR International, Radnor, PA, USA). Chemiluminescence was quantified on a GloMax® plate reader (luminescence module; Promega) every 2 min for 30 min with a 1000 ms integration time.

Flg22‐induced MITOGEN‐ACTIVATED PROTEIN KINASE (MAPK) activation was assessed (Xu *et al*., 2018) using leaf disks collected from six plants per CO_2_ treatment. Leaf disks were maintained in CO_2_ chambers, treated with 10 μM of flg22 peptide, and sampled between 0 and 250 min. Total protein was extracted and normalized (Wang *et al*., 2018), and MAPK activation was assessed by immunoblot analysis using primary anti‐Phospho‐p44/42 MAPK antibody (Erk1/I 2; Thr‐202/Tyr‐204) (Cell Signaling, Danvers, MA, USA) and secondary goat anti‐rabbit‐HRP antibody (Cell Signaling).

### Bacterial infection assays

*Pseudomonas syringae* pv. *glycinea* (*Psg*) race 4, *Pst*DC3000, and *Pst*DC3000 *hrcC‐* (*hrcC‐*) were grown at 30°C overnight in Luria Bertani (LB) broth with appropriate antibiotics. The next day, cells were harvested and resuspended in 10 mM MgCl_2_ at OD_600_ = 0.2 (*c*. 1 × 10^8^ colony‐forming units (CFU) ml^−1^). Immediately before inoculations, 0.04% of Silwet L‐77 (ThermoFisher Scientific) was added to the suspension, and the unifoliate leaves of 14‐d‐old soybeans were spray‐inoculated on adaxial and abaxial surfaces until fully wet. Leaf disks were extracted from 24 plants per treatment, between 1‐ and 7 d post inoculation (dpi). Leaf disks from three plants were pooled per sample and were used to quantify CFU cm^−2^ (Liu *et al*., 2015). For phytohormone analyses, eight plants per treatment were sampled at 6‐ and 24 h post inoculation (hpi) and 3′ mRNA sequencing (3′ mRNA‐Seq) analysis was performed using 6 hpi samples. Detailed methods describing extraction and quantification of the phytohormones SA and JA are provided in Methods S1. *g*_sw_ in response to *Psg* was measured at 1 hpi using 10 plants per treatment. For 3′ mRNA‐Seq analyses, leaves were collected from eight *Psg*‐infected and mock‐inoculated plants per CO_2_ treatment and chamber, yielding three biological replicates per treatment (96 samples, QuantSeq dataset 2). Samples were collected between 09:00 h and 10:00 h for all replicates.

### Bacterial growth curves

Liquid *Pst*DC3000 cultures were seeded at a concentration of 1 × 10^7^ CFU ml^−1^ in 100 ml of LB broth containing appropriate antibiotics. Cultures were grown in 500‐ml Erlenmeyer flasks with gas‐permeable caps to allow free diffusion of CO_2_ and kept in temperature‐controlled growth chambers maintained at 28°C (no light), *c*. 20% relative humidity, and either 419 or 550 ppm of CO_2_. Cultures were shaken at 100 rpm and OD_600_ was recorded every 4 h over 32 h.

### QuantSeq 3′ mRNA‐Seq analyses

Total RNA was extracted from *c.* 50 mg of soybean leaf tissue using the Direct‐zol RNA Miniprep Plus Kit with DNaseI treatment (Zymo Research, Irvine, CA, USA). RNA samples were quantified using a Qubit 4.0 Fluorometer (Invitrogen), and RNA integrity was assessed using the Fragment Analyzer Automated CE System. All RNA samples used for sequencing had an RQN (RNA Quality Number) equal to or > 7. Library preparation was performed using the QuantSeq 3′ mRNA‐Seq Library Prep Kits (Lexogen, Greenland, NH, USA), and samples were sequenced using the Illumina NovaSeq 6000 System (Iowa State University DNA Facility). Details of bioinformatic and statistical analyses of 3′ mRNA‐Seq data to identify differentially expressed genes (DEGs) and subsequent analysis of DEG annotation and identification of overrepresented transcription factors and promoter elements are provided in Methods S1.

### Viral infection assays

Lyophilized soybean leaves infected with BPMV or SMV were ground in 10 ml of 50 mM potassium phosphate buffer (pH 7.5) per 1 g of tissue. One unifoliate leaf of 14 dap soybean was dusted with carborundum (Fisher Scientific) and rub‐inoculated with 50 μl of inoculum or buffer (Mock). The second unifoliate leaf was inoculated 2 d later using the same method. Systemic leaves were sampled from six plants per treatment, at 14 and 21 dpi to assess viral titer. Fresh weight, dry weight, and gene expression were measured using 21 dpi plants. Disease severity was evaluated between 0 and 21 dpi using a single‐digit disease rating scale ranging from 0 to 3 (0–no disease, 1–mild, 2–moderate, and 3–severe). Area under disease progress curve (AUDPC) was calculated based on the equation (Simko & Piepho, 2012).

$$\text{AUDPC}=\sum_{i=1}^{n-1}\left[\frac{D_{\left(i+1\right)}+D_{i}}{2}\right]\left[T_{\left(i+1\right)}-T_{i}\right]$$

$D_{i}$, disease severity on *i* ^th^ date; $T_{i}$, *i* ^th^ date; n, total number of observations.

### *Fusarium virguliforme* and *P. sylvaticum* infection assays

Sterilized sorghum (*Sorghum bicolor* (L.) Moench) seeds were inoculated with *F. virguliforme* O'Donnell and T. Aoki (isolate LL0036) and incubated in the dark at room temperature for 21 d and dried for 3–4 d. Styrofoam cups (8 oz.) were filled with a mixture of 180 ml of sterile sand soil and 9 ml of *F. virguliforme*‐infested sorghum. Soybean seeds were placed in the cups and covered with 25 ml of peat mix. At 35 dap, roots and shoots were sampled from six cups of each CO_2_ treatment to measure fresh weight and dry weight. Disease severity was assessed on leaves on a 0 to 7 rating scale (Roth *et al*., 2019) (Table S2), and the AUDPC was calculated. For plate growth assays, a starting culture of *F. virguliforme* was prepared on 1/3 strength potato dextrose agar (PDA) and grown at room temperature for 21 d in the dark. A 7‐mm plug was punched out of the starting culture and placed into the center of a 90‐mm‐diameter Petri dish containing 1/3 strength PDA. Ten plates per CO_2_ treatment were maintained in the dark under *a*CO_2_ or *e*CO_2_, and hyphal growth was assessed every other day for 11 d.

Millet was inoculated with a 3‐d‐old culture of *P. sylvaticum* W. A. Campb. & F. F. Hendrix 1967 and incubated in the dark at room temperature for 7–14 d (Matthiesen *et al*., 2016). A mixture of 100 ml of sterile sand soil and 5 ml of *P. sylvaticum*‐infested millet was placed in a 237 ml (8 oz.) polystyrene cup and covered with 25 ml of peat mix. The seed was placed on top of the peat mix and then covered with another 25 ml layer of peat mix. At 14 dap, plants in six cups of each treatment were uprooted, roots were washed, and disease severity was assessed using a 0 to 4 rating scale (Zhang & Yang, 2000) (Table S3). Root and shoot fresh weight and *P. sylvaticum* copy numbers were determined at 21 dap. For plate growth assays, a starting culture of *P. sylvaticum* (isolate Gr8) was grown on 1/2 strength PDA at room temperature for 5 d in the dark. A 7‐mm plug was punched and transferred into the edge of a 90‐mm‐diameter Petri dish containing 1/2 strength PDA. Ten plates per treatment were incubated in the dark under *a*CO_2_ or *e*CO_2_, and growth was measured from the edge of the inoculum plug to the tip of the longest hypha every 24 h for 96 h.

### Statistical analysis

Linear mixed‐effects models (LMM) were fit to each response following the experimental design. Taking the bacterial infection experiment measuring CFUs as an example, the whole‐plot factor was CO_2_ level and the whole‐plot experimental units were growth chambers. Randomized complete block design was implemented at the whole‐plot level, with each block comprising a pair of chambers at *a*CO_2_ or *e*CO_2_. At the split‐plot level, 24 plants were assigned to each pathogen, with sets of three plants pooled for each CFU measurement, repeated over time. Following this split‐plot design, the fixed effects of the LMM include the main effects and all interaction effects of the factors CO_2_, bacterial species, and time, and random effects include block, chamber, split‐plot, and sets of plants. Similar to this example, LMMs were fit for other responses following the corresponding experimental designs.

For response variables that exhibited unequal variances on the original scale, LMM analysis was done on power‐ or log‐ transformed data, and the Delta method was used to compute SE for the original scale. For all LMM analysis, model diagnostic checks were done to ensure that model assumptions were appropriate. Parameters of LMM were estimated using lmer() in the lme4 R package. The emmeans() function in the emmeans R package was used to compute the SE. Type III ANOVA tables with *F*‐tests were conducted by anova() and the lmertest R package.

## Results

### Elevated CO_2_ alters soybean physiology and gene expression

**Fig. 1:**
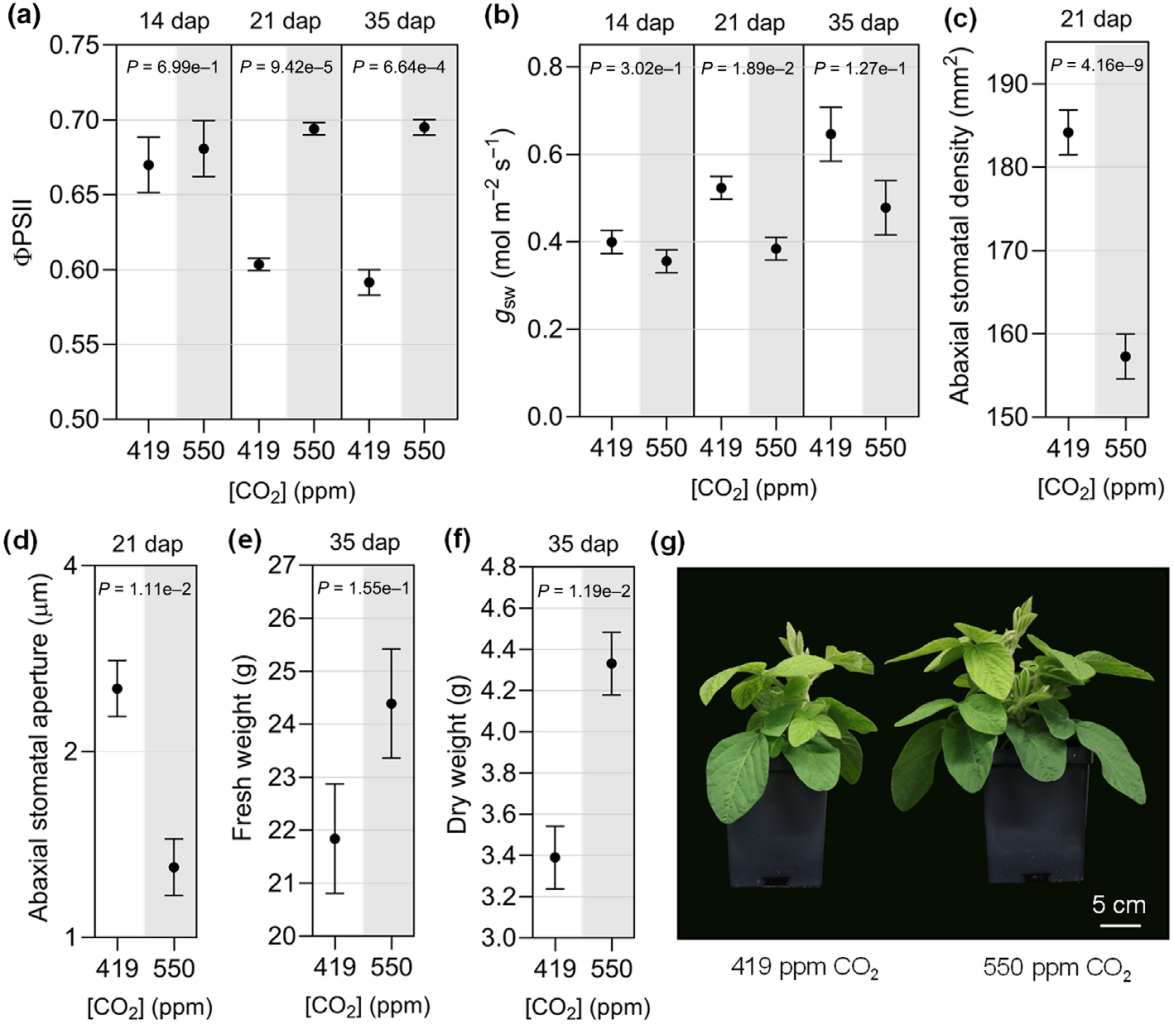
Effects of elevated CO_2_ on soybean physiology and growth. (a) Photosystem II (ΦPSII) activity and (b) stomatal conductance (*g*_sw_) were measured at the indicated days after planting (dap) using a LI‐600 portable system. (c) Stomatal density and (d) stomatal aperture measured at 21 dap using three randomly selected fields of view per leaf sample using a brightfield microscope. (e) Shoot fresh weight, (f) shoot dry weight, and (g) representative photos of soybean plants at 35 dap. Measurements and samples were taken from the unifoliate leaves at 14 dap or the newest fully expanded trifoliate leaves at 21 and 35 dap of 10 plants at each time point, except ΦPSII and *g*_sw_, which were measured in 15 plants per CO_2_ treatment. The three experimental replicates were conducted simultaneously in independent CO_2_ controlled chambers using a replicated completely randomized design. Data were graphed as the mean across the three replicates with SE bars. Linear mixed effect model (LMM) analysis was applied to the fourth power of ΦPSII 35 dap and log‐transformed stomatal aperture data due to unequal variance on the original scale. *P*‐values were computed based on *F*‐tests for the effect of CO_2_ at each time point from the LMM analysis. The letter e denotes an exponent to the power of 10 and ppm denotes parts per million.

Before studying the effects of *e*CO_2_ on soybean defense, we wanted to ensure that our growth conditions caused changes in physiological responses that were previously observed under higher atmospheric CO_2_ (Ainsworth & Long, 2021; Li *et al*., 2021). At 14 dap, unifoliate leaves of soybean plants grown in *e*CO_2_ displayed no difference in ΦPSII activity compared with plants grown at *a*CO_2_ (Fig. 1a). However, at 21 and 35 dap, ΦPSII activity was higher in *e*CO_2_ (Fig. 1a), indicating a higher photosynthetic rate in trifoliate leaves. Stomatal conductance to water vapor (*g*_sw_) was also affected, with trifoliate leaves of plants grown at *e*CO_2_ displaying lower *g*_sw_ at 21 and 35 dap (Fig. 1b), which was consistent with the lower stomatal density (Fig. 1c) and reduced stomatal aperture (Fig. 1d) on the abaxial leaf surface. In line with the lower stomatal density, a reduction in stomatal index was observed at *e*CO_2_, indicating that the decrease in stomatal density was due to reduced stomatal production and not an increase in leaf expansion (Fig. S2). At 35 dap, soybeans grown in *e*CO_2_ had greater shoot fresh weight (Fig. 1e) and dry weight (Fig. 1f) and were visibly larger than those grown in *a*CO_2_ (Fig. 1g). Together, these experiments demonstrate that a 31% increase in [CO_2_] induced physiological responses under our conditions that were consistent with prior studies.

**Fig. 2:**
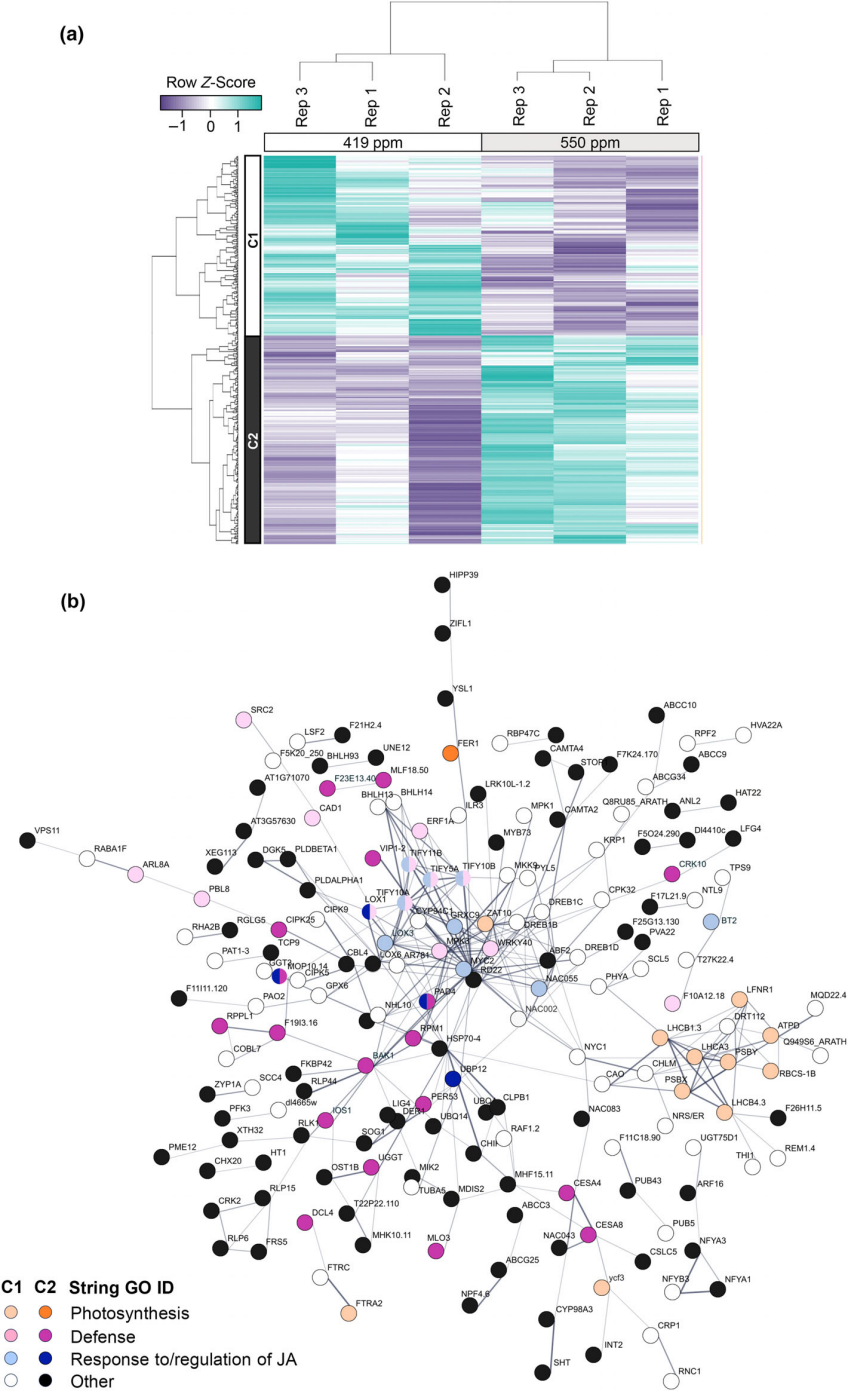
Transcriptomic analysis of soybean gene expression in leaves of plants grown under ambient CO_2_ (*a*CO_2_) (419 parts per million (ppm)) or elevated CO_2_ (*e*CO_2_) (550 ppm). (a) Differentially expressed genes (DEG) responding to *e*CO_2_ were identified using a false discovery rate < 0.01 (Supporting Information Table S4). Samples for 3′ mRNA‐Seq analysis were taken from the newest fully expanded trifoliate leaves of soybean plants at 21 d after planting (dap). The three experimental replicates (Rep) were conducted simultaneously in independent CO_2_‐controlled chambers using eight plants per CO_2_ treatment. Row *Z*‐scores were used for hierarchical clustering of DEGs, based on expression across samples and replicates. Two expression clusters were identified. DEGs in Cluster 1 (C1) were expressed at higher levels in 419 ppm vs 550 ppm CO_2_ and DEGs in Cluster 2 (C2) were expressed at higher levels in 550 ppm vs 419 ppm CO_2_. (b) STRING network for DEGs identified between *a*CO_2_ and *e*CO_2_ in leaves at 21 dap. The 388 genes identified in soybean correspond to 321 genes in *Arabidopsis thaliana* that were used to assign functional annotations (String Gene Ontology Identifiers (GO ID)). Lightly shaded or white circles indicate DEGs from C1 and brightly shaded and black circles indicate DEGs from C2.

To investigate gene expression changes associated with physiological responses to *e*CO_2_, we conducted 3′ mRNA‐Seq analysis using RNA isolated from trifoliate leaves at 21 dap. We identified 388 DEGs (Table S4), which were used to construct a heat map that formed two distinct clusters associated with [CO_2_] (Fig. 2a). The 180 DEGs in Cluster 1 were repressed in *e*CO_2_, while the 208 DEGs in Cluster 2 were induced in *e*CO_2_ (Table S4). To identify gene networks responding to [CO_2_], the 321 best BLASTP Arabidopsis homologs corresponding to our 388 soybean DEGs were were used as input for STRING (Szklarczyk *et al*., 2023) and Gene Ontology (GO) analyses (Szklarczyk *et al*., 2023) to identify biological processes (BP) that were overrepresented within each cluster (Table S5). In Cluster 1, two major themes emerge related to: responses to JA signaling (e.g. response to wounding, response to hormone, regulation of defense response, response to lipid, and regulation of JA‐mediated signaling); and photosynthesis (e.g. response to light intensity, response to high light intensity, photosynthesis, and photosynthesis light reaction). The STRING association network produced from the genes in Cluster 1 also highlights the effects on JA signaling with a node of highly connected genes that include *MYC2* transcription factor and *TIFY* (JAZ repressor) genes (Fig. 2b, light blue). A node of highly connected genes in the lower right quadrant includes several genes related to photosynthetic processes, including soybean homologs of *AtRbcS1B*, *AtRAF1*, *AtLHCB8*, *AtLHCB1*, *AtCHLM*, and *AtPIF1* (Fig. 2b, light orange).

Major themes emerging from Cluster 2 are BP terms related to: defense (e.g. defense, response to external stimulus, response to biotic stimulus, response to bacterium, and response to other organism); and protein modification (e.g. protein phosphorylation, protein modification process, and phosphorus metabolic process) (Table S5). The STRING association network identifies interconnected genes associated with defense responses (Fig. 2b). These nodes include soybean homologs of *AtBAK1*, *AtPAD4*, and *AtIOS1* (Table S4), all required for pattern‐triggered immunity (PTI) responses (Yeh *et al*., 2016; Pruitt *et al*., 2021), and a number of resistance‐like proteins, including *AtRPM1*, *AtRLP1*, and *AtRLP44*. While not highlighted in the STRING analyses, soybean homologs of genes associated with reduced *g*_sw_ and stomatal aperture (*AtHT1*, *AtFER*, *AtZIFL1*, *AtPLDa1*, and *AtNRT1:2*) were upregulated in *e*CO_2_ (Table S4). Together, our gene expression analyses indicate that *e*CO_2_ causes changes in the transcriptome of unifoliate soybean leaves, repressing expression of photosynthetic genes, altering JA signaling, priming innate immunity against microbial pathogens, and promoting expression of genes associated with stomatal closure.

### Soybean resistance to *Pseudomonas* spp. is enhanced under *e*CO_2_

**Fig. 3:**
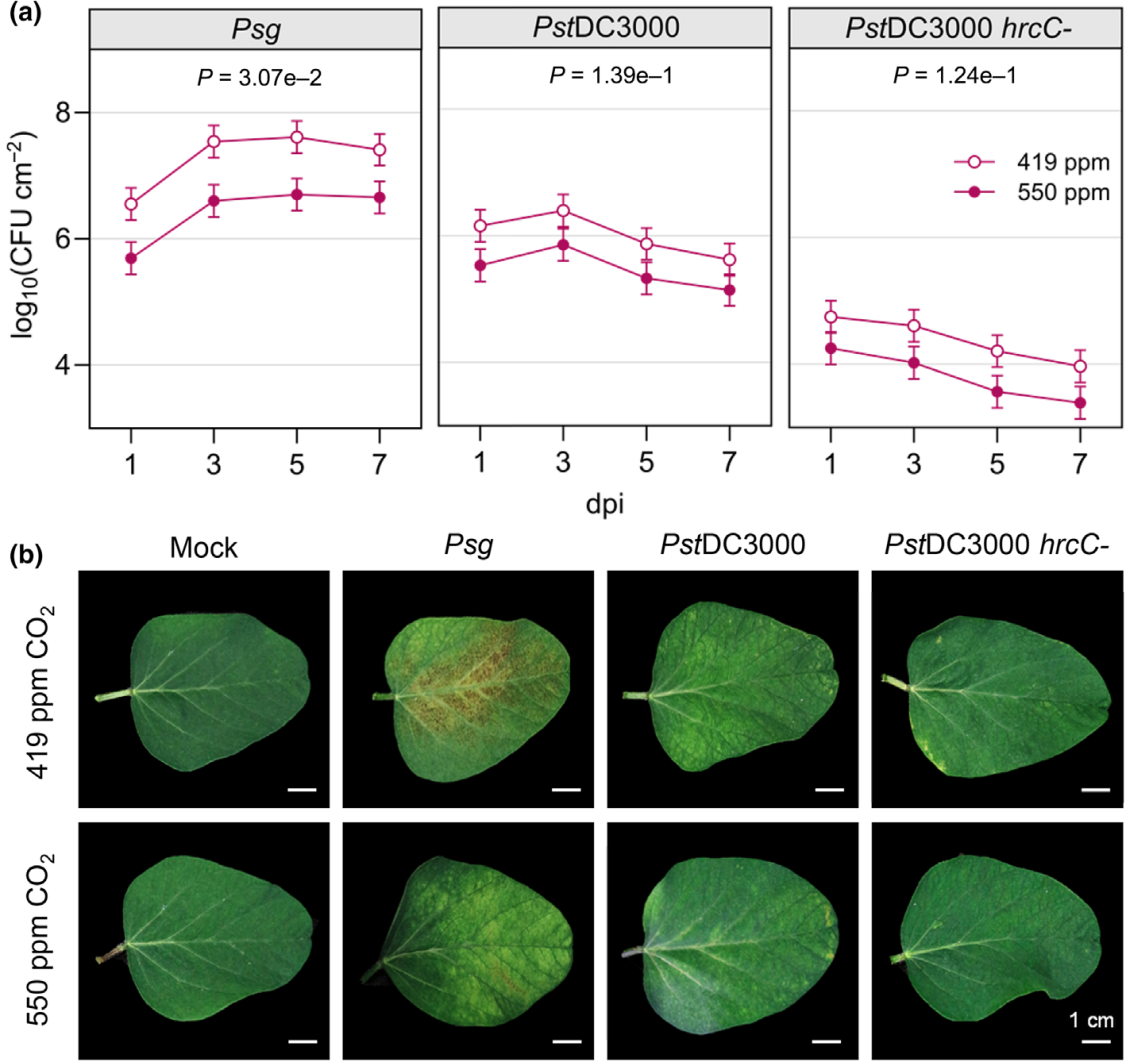
Soybean plants growing in elevated CO_2_ are less susceptible to *Pseudomonas syringae*. (a) Colony‐forming units (CFU) of *P. syringae* pv. *glycinea* race 4 (*Psg*), *P. syringae* pv. *tomato* (*Pst*DC3000) and *Pst*DC3000 *hrcC‐* were quantified in the unifoliate leaves of plants at 1, 3, 5, and 7 d post inoculation (dpi). The unifoliate leaves of 24 plants were sampled for each treatment group, and three independent biological replicates were performed. Data points represent the mean CFU count of the three biological replicates with SE bars. *P*‐values were computed using *t*‐tests for the contrast between two CO_2_ levels of each pathogen averaged over dpi from the linear mixed effect model analysis. (b) Representative images of unifoliate leaves photographed at 7 dpi. Mock plants were treated with 10 mM MgCl_2_, 0.04% Silwet L‐77 solution with no bacteria. The letter e denotes an exponent to the power of 10.

In order to assess the impacts of *e*CO_2_ on susceptibility to microbial pathogens, we first used the well‐characterized soybean–*P. syringae* pathosystem (Lindeberg *et al*., 2009; Whitham *et al*., 2016). Basal defense against bacterial pathogens is initiated by cell surface receptors that recognize microbe‐associated molecular patterns (MAMPs), such as the bacterial flagellin peptide flg22 (DeFalco & Zipfel, 2021). *Psg*, which causes bacterial blight in soybean, was inoculated on the unifoliate leaves of 14‐d‐old plants and chosen to model a compatible interaction where effectors effectively suppress immunity resulting in disease (Prom & Venette, 1997). At 1 dpi, the proliferation of *Psg* was 1.1‐log_10_ fold lower in plants grown in *e*CO_2_ compared to *a*CO_2_ (Fig. 3a), and it remained significantly reduced in *e*CO_2_ over the 7‐d time course (Fig. 3a). In compatible interactions, *Psg* infection causes the development of necrotic spots that become surrounded by yellow halos (Budde & Ullrich, 2000). At 7 dpi, soybeans infected under [*a*CO_2_] displayed necrotic lesions across the surface of leaves, consistent with bacterial blight symptoms (Fig. 3b). By contrast and consistent with reduced *Psg* growth, few disease symptoms were observed on the leaves of plants grown at *e*CO_2_ (Fig. 3b).

We also inoculated the unifoliate leaves of 14‐d‐old plants with *Pst*DC3000, as an example of an incompatible interaction causing hypersensitive cell death resulting in disease resistance (Kobayashi *et al*., 1989), and the *Pst*DC3000 type III secretion mutant, *hrcC‐*, that cannot secrete bacterial effector proteins and therefore cannot multiply or induce hypersensitive cell death (Brooks *et al*., 2004). Lower CFUs were consistently observed across the time course in soybean grown at *e*CO_2_ when inoculated with either *Pst*DC3000 or *hrcC‐*, although the *P*‐values were > 0.05 (Fig. 3a). At 7 dpi, *Pst*DC3000‐infected plants grown at either [CO_2_] began to show signs of localized chlorosis and cell death while *hrcC‐* infection did not result in obvious disease or defense phenotypes, regardless of the [CO_2_] (Fig. 3b).

To assess whether atmospheric [CO_2_] independently affects bacterial growth rate, we conducted a growth curve analysis on liquid cultures of *Pst*DC3000 in our controlled environment growth chambers. The growth curves of *Pst*DC3000 cultures were similar between the two CO_2_ levels (Fig. S3), indicating that reduced growth of *P. syringae* in soybean is not due to direct effects of *e*CO_2_ on bacterial multiplication. Together, these data suggest that *e*CO_2_ enhances resistance to *P. syringae* infection in soybean during compatible interactions and, to a lesser extent, during an incompatible interaction.

### Elevated CO_2_ alters basal immune responses

**Fig. 4:**
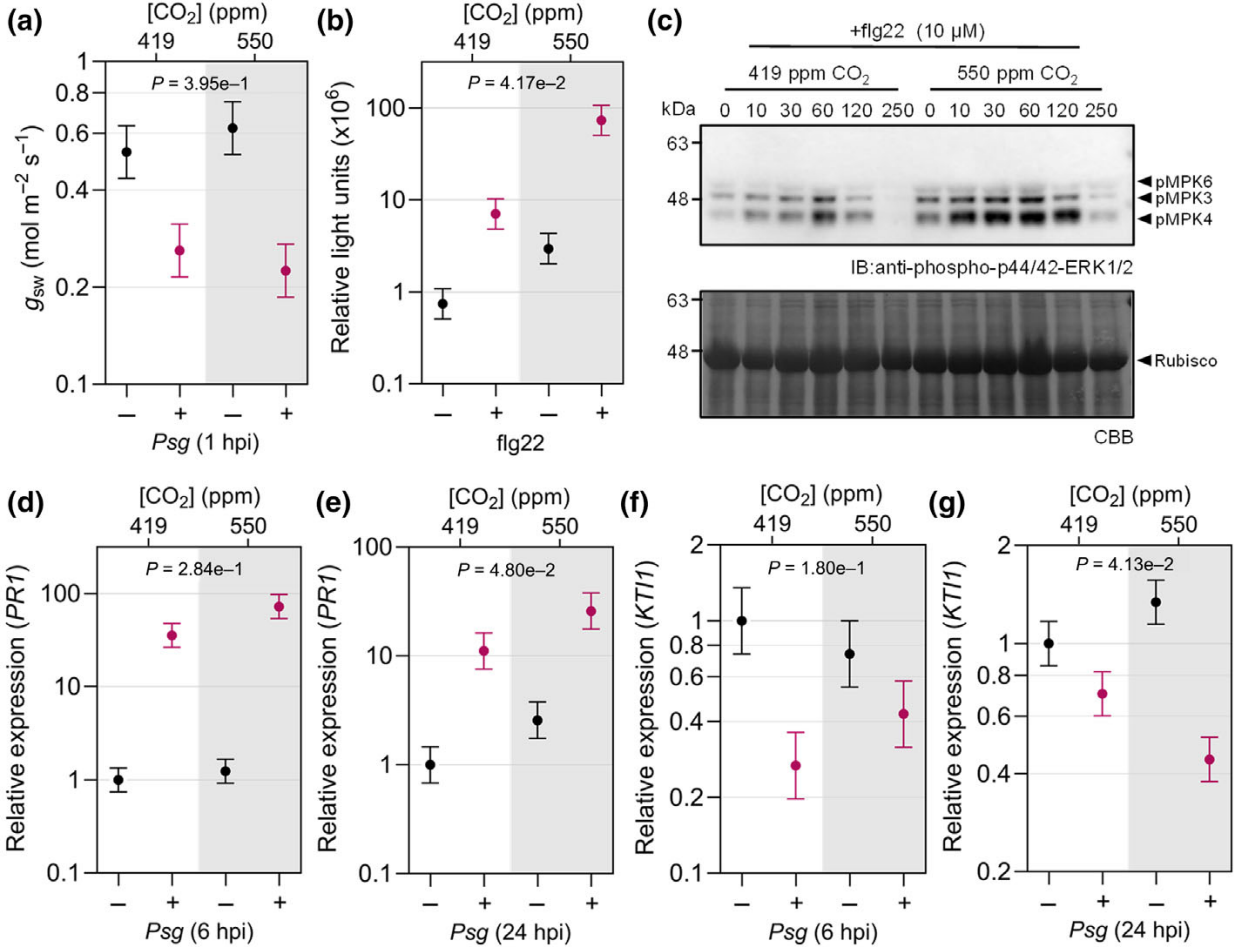
Bacterially induced defense signaling is more strongly upregulated in soybean leaves grown under elevated CO_2_. (a) Stomatal conductance (*g*_sw_) at 1 h post inoculation (hpi) with *Pseudomonas syringae* pv. *glycinea* race 4 (*Psg*). (b) Reactive oxygen species (ROS) production in response to bacterial flagellin 22 (flg22) peptide was quantified using a chemiluminescence assay. Relative light units were determined using leaf disks from 12 plants per treatment, and (c) flg22‐induced MAPK activation using protein extracted from 12 plants per time point (0 to 250 min after treatment) and visualized by immunoblot analysis. Expression of *PR1* (SA marker gene) (d) 6 hpi and (e) 24 hpi and expression of *KTI1* (JA marker gene) (f) 6 hpi and (g) 24 hpi with *Psg* or mock treatment was assessed by reverse transcription quantitative polymerase chain reaction. RNA was extracted from six plants per treatment, and expression was measured relative to the *Skp1* housekeeping gene. All experiments were conducted using leaves or leaf disks collected from 14‐d‐old plants grown under 419 parts per million (ppm) or 550 ppm CO_2_. Three independent replicates were conducted for each experiment. Data points represent average values across the three experimental replicates with SE bars. *P*‐values for ROS production and *PR1* expression were computed using *F*‐tests for the main effect of CO_2_, and *g*_sw_ and *KTI1* expression using the interaction effect between CO_2_ and *Psg*, from the linear mixed effect model analysis based on log‐transformed data. The letter e denotes an exponent to the power of 10.

To determine whether *e*CO_2_ affects immunity to *Psg* at a molecular level, we assessed multiple hallmarks of plant immune signaling. In response to *Psg*, the change in stomatal conductance following *Psg* treatment did not differ between [CO_2_] (Fig. 4a). However, soybeans grown at *e*CO_2_ exhibited greater overall production of reactive oxygen species when considering both mock‐ and flg22‐treated leaf disks (Fig. 4b) as well as higher and more sustained MAPK activation (Fig. 4c). We also observed that a soybean *PR1* homolog, an SA‐regulated defense gene, accumulated to higher levels in mock‐ and *Psg*‐inoculated plants grown in *e*CO_2_, particularly in the 24 hpi sample (Fig. 4d,e) and corresponded to a slightly greater SA accumulation in leaves sampled at 6 hpi (Fig. S4a). At 24 hpi, expression of the JA marker gene *KTI1* was more downregulated in response to *Psg* (Fig. 4f,g). However, JA accumulation was not affected by [CO_2_] in our metabolite analysis (Fig. S4b). Together, these data suggest that bacterially induced immune signaling is more robust in plants grown in *e*CO_2_, which correlates with the enhanced resistance to *Pseudomonas* spp. in these plants.

### *e*CO_2_ alters the soybean transcriptome in response to *Psg*

To gain further insight into the effects of *e*CO_2_ on molecular responses to *Psg* treatment, we conducted 3′ mRNA‐Seq analysis on the unifoliate leaves at 6 hpi with buffer alone (mock) or with *Psg*. We identified DEGs in mock‐ vs *Psg‐*treated plants grown at each of the two [CO_2_]: 18054 DEGs were identified in plants grown in *a*CO_2_ and 19 443 DEGs were identified for plants grown in *e*CO_2_. We produced a combined list of all unique DEGs for a total of 21 822 *Psg‐*responsive DEGs, which was used to generate a heat map that formed four distinct clusters (Fig. S5; Table S6). Cluster 1 (1661 DEGs) had mixed expression in response to *Psg* treatment and no significantly (corrected *P* < 0.05) overrepresented GO terms (Table S7). Cluster 2 (9822 DEGs) was induced by *Psg* treatment and had 81 significant GO terms associated with stress (response to cadmium ion, heat, hypoxia, oxidative stress, salt, and UV‐B), defense (response to chitin, defense response to bacterium, fungus, and virus, cell death, regulation of SA biosynthesis, response to SA, hypersensitive response, and systemic acquired resistance), and ER stress (protein folding, response to unfolded protein, and response to ER stress). Clusters 3 (6718 DEGs) and 4 (3621 DEGs) were repressed by *Psg* treatment. While Cluster 3 had 50 significant GO terms associated with photosynthesis, response to light, and circadian rhythm, Cluster 4 had two significant GO terms: response to starvation and oxidation–reduction process. Among DEGs repressed by *Psg* treatment in *a*CO_2_ and *e*CO_2_ conditions, 61.6% had greater negative fold‐changes in *e*CO_2_ (7419/12 053 DEGs). Similarly, for DEGs induced by *Psg* in *a*CO_2_ and *e*CO_2_ conditions, 60.1% had greater positive fold‐changes in soybeans grown in *e*CO_2_ (5671/9357 DEGs).

**Fig. 5:**
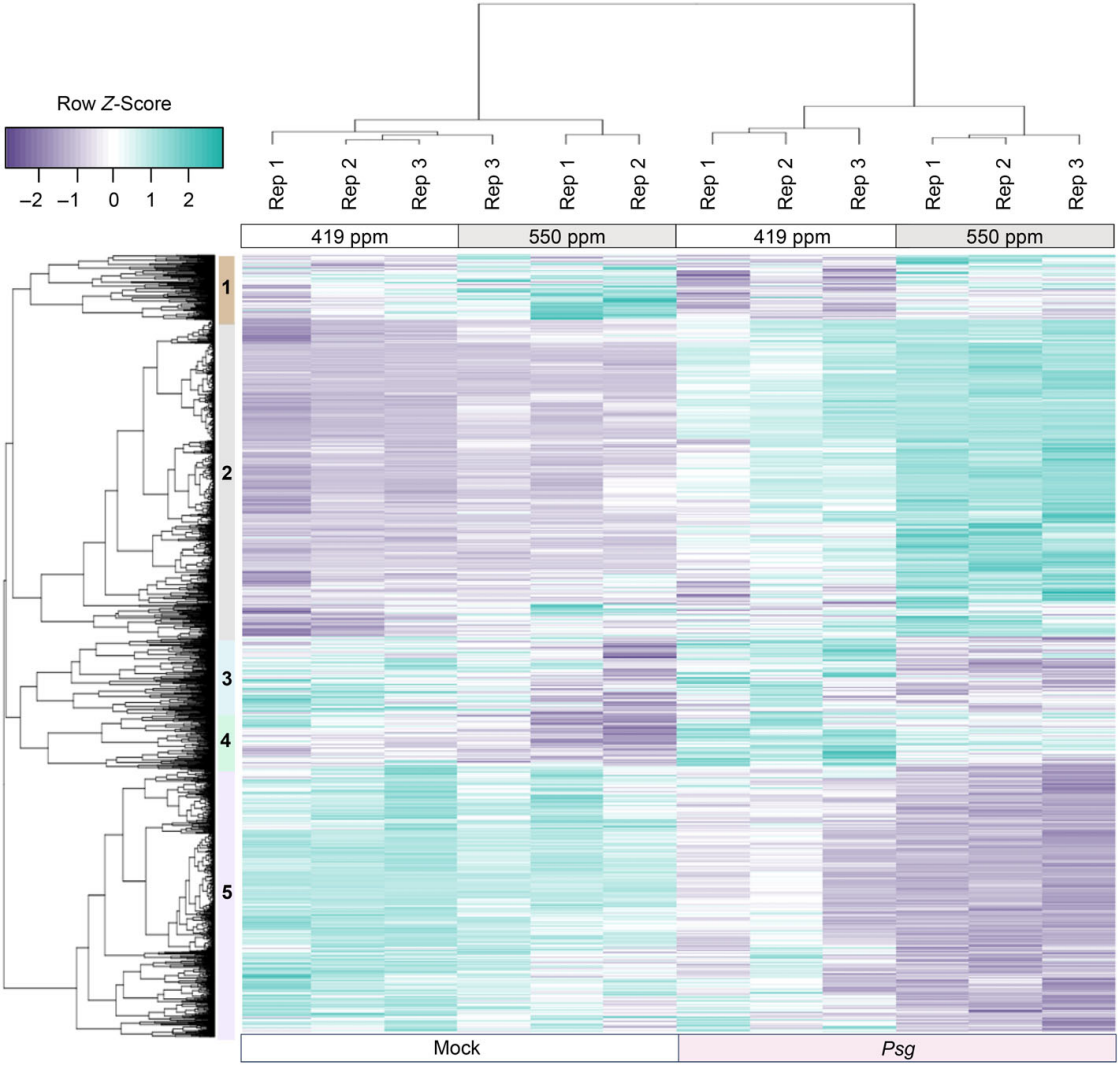
Identification of soybean differentially expressed genes (DEGs) responding to elevated CO_2_ (*e*CO_2_) (419 parts per million (ppm) vs 550 ppm) in either mock or *Pseudomonas syringae* pv. *gylcinea* (*Psg*)‐infected samples at 6 h post inoculation (hpi). Samples for 3′ mRNA‐Seq analysis were taken from the unifoliate leaves of 14‐d‐old plants at 6 hpi with mock treatment or *Psg*. The three independent biological replicates (Rep) were conducted using eight plants per CO_2_ treatment. Using a false discovery rate < 0.01, we identified 2419 differentially expressed genes (DEG) responding to *e*CO_2_ in *Psg* and/or mock‐treated samples. Row *Z*‐scores were used for hierarchical clustering of DEGs, based on expression across samples and replicates. Purple indicates expression values below the row mean for a given DEG and sample and teal indicates expression values above the row mean for a given DEG and sample. Rep indicates the independent biological replicate. The five major expression clusters of DEGs are indicated as 1 to 5, with Clusters 2 and 5 containing the most genes. Cluster 2 contains 986 genes that are primarily upregulated at 6 hpi with *Psg* but these genes are upregulated more in *e*CO_2_. Cluster 5 contains 828 genes that are downregulated at 6 hpi with *Psg*, but these genes are more downregulated in *e*CO_2_.

To further understand how responses to *Psg* differ with changes in atmospheric [CO_2_], we identified 622 and 1919 DEGs associated with *e*CO_2_ vs *a*CO_2_ in mock‐ or *Psg*‐inoculated plants, respectively (Table S8). Using a union of these two lists, we generated a heatmap of the 2419 DEGs, which formed five distinct clusters (Fig. 5). To assign potential function to each of the clusters, we used the Arabidopsis best blastp homologs corresponding to all DEGs within a cluster as input into STRING (Szklarczyk *et al*., 2023) and performed GO BP enrichment for each cluster (Table S9). Cluster 1, which tended to be induced by *e*CO_2_ in mock and *Psg* samples, was associated with 40 GO terms related to stress (response to chemical, stress, and abiotic stimulus) and defense (regulation of JA signaling, response to JA, response to bacterium, and defense response). DEGs in Cluster 2 were induced by *Psg* with greater induction observed in *e*CO_2_ and corresponded to 139 significant GO terms related to stress and defense responses. DEGs in Cluster 3 were repressed by *e*CO_2_ and corresponded to 37 significant GO terms, many associated with stress and regulation (regulation of transcription, circadian rhythm, primary metabolism, development, and photoperiodism). DEGs in Cluster 4 were induced by *Psg* with greater induction observed in *a*CO_2_ and corresponded with 83 significant GO terms associated with stress, signaling, development, and defense. DEGs in Cluster 5 were repressed by *Psg*, with greater repression at *e*CO2. The 37 GO terms in Cluster 5 were largely associated with photosynthesis and response to light.

Across all 2419 DEGs, 74.8 and 83.2% of DEGs responding to *e*CO_2_ in *Psg* or mock were also significantly differentially expressed in response to *Psg* at *a*CO_2_ and *e*CO_2_, respectively. This confirms that *e*CO_2_ impacts the expression of genes involved in pathogen defense responses. This is most obvious in Clusters 2 (986 DEGs) and 5 (828 DEGs), where 71.1 and 94.3% of DEGs are also significantly differentially expressed in response to *Psg* at *a*CO_2_ and 82.3 and 99.5% of DEGs are also significantly *Psg*‐responsive in *e*CO_2_. In summary, the 3′ mRNA‐Seq analyses indicate that *e*CO_2_ does not cause a reprogramming of responses to *Psg*, but rather it enhances the up‐ or downregulation of many genes associated with defense and stress responses and photosynthetic processes.

### Identification of regulatory elements controlling response to *e*CO_2_

We next sought to identify the transcription factors (TFs) that were differentially expressed in the 2419 DEGs responding to *e*CO_2_ in Fig. 5. Across all five clusters, we identified 144 TFs representing 31 transcription factor families. Clusters 1–5 contained 7, 43, 27, 19, and 48 unique TFs, respectively (Table S10). Given that many of the GO term descriptions significantly overrepresented in Clusters 1 through 5 included the words ‘response to’ or ‘regulation of’, we were interested in predicting upstream TFs that could also be important in *e*CO_2_ responses. Using the DEG list for each cluster as input into PlantRegMap (Tian *et al*., 2020), we identified 1, 44, 18, 13, and 16 significantly overrepresented TF binding sites (TFBS) for Clusters 1–5, respectively (Table S11). Interestingly, some predicted TFBS were significant in multiple clusters. In addition, five predicted TFBS were also among our DEGs of interest. We identified the DEG targets corresponding to each overrepresented TFBS (Table S12). In order to assign a possible function to a TF with an overrepresented TFBS, we used the SoyBase GO Term Enrichment Tool (https://www.soybase.org/goslimgraphic_v2/dashboard.php) to identify significantly overrepresented GO terms (corrected *P* < 0.05) associated with the corresponding target DEGs (Table S13). Of the 92 significant TFBS predicted, 48 had significantly overrepresented GO terms. Identified GO terms were associated with stress (response to heat, H_2_O_2_, redox state, phosphate starvation, and light), regulation (cell aging, circadian rhythm, growth rate, proton transport, and transcription), photosynthesis (light reaction, electron transport, and plastid organization), and defense (fatty acid elongation, biosynthesis of anthocyanins, carotenoids, and flavonoids, and negative regulation of defense response to bacterium). Sorting the data in Table S13 revealed multiple TFs were regulating DEGs corresponding to the same GO term. For example, we identified nine significant TFBS associated with photosynthetic electron transport in photosystem I.

**Fig. 6:**
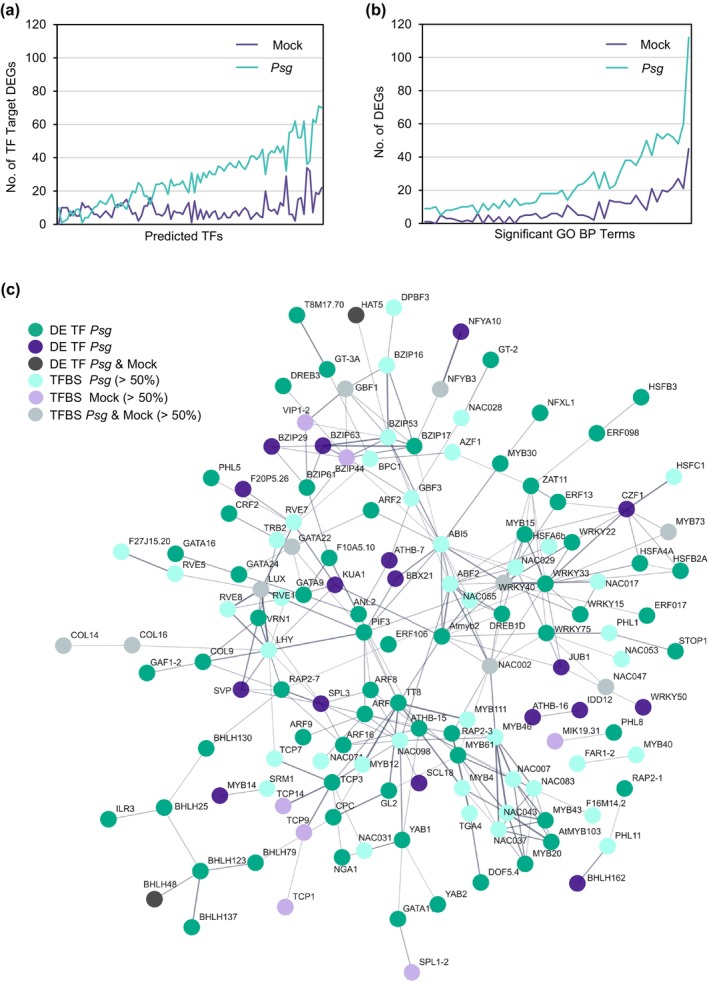
Significantly differentially expressed transcription factors (TF) and overrepresented TF binding sites (TFBS) are more robust in *Pseudomonas syringae* pv. *glycinea* (*Psg*)‐infected tissues responding to elevated CO_2_ (*e*CO_2_) than mock‐inoculated tissues. PlantRegMap/PlantTFDB v.5.0 (Tian *et al*., 2020) was used to identify significantly differentially expressed TFs and significantly overrepresented TFBS among the differentially expressed gene (DEG) clusters identified in Fig. 5 (Supporting Information Tables S9–S11). (a) Significant TFBS vs predicted number of DEG targets. Overrepresented TFBS and DEG targets are plotted for every cluster (cluster information not shown); therefore, if a TFBS was significant in multiple clusters, it was plotted multiple times but with different DEG targets. (b) Significantly overrepresented Gene Ontology (GO) terms (minimum of 10 DEGs per GO term) vs DEG number (Table S5). In both panels, DEG in teal were significant in *Psg* samples and/or DEG in purple were significant in mock‐treated samples. (c) Best Arabidopsis homologs of TFs and TFBS were input into STRING (Szklarczyk *et al*., 2023). TFs differentially expressed in response to *e*CO_2_ in *Psg*‐treated samples, mock‐treated samples or both are colored dark teal, dark purple or gray, respectively. TFBS are colored based on the origin of the target DEG.

Given these results, we wanted to examine the TF/TFBS data relative to our DEGs from mock and *Psg*‐infected tissues. We plotted the number of target DEGs corresponding to each predicted TFBS (Fig. 6a). For almost every predicted TFBS, there were more targets among DEGs from *Psg*‐infected tissues than mock‐inoculated tissues, suggesting *Psg* infection resulted in a more robust response to *e*CO_2_. To confirm this pattern was not due to errors in predicting TFBS, we also plotted the number of DEGs associated with significantly overrepresented GO terms (Fig. 6b; Tables S8, S9). For almost every significant GO term, there were more GO terms associated with *Psg*‐infected tissues than mock‐inoculated tissues. To better visualize the interaction between TF and TFBS, we used the best Arabidopsis homolog of each TF and TFBS as input into STRING (Szklarczyk *et al*., 2023). In Fig. 6c, TF and TFBS associated with *Psg*‐infected tissues are colored shades of teal, while TF and TFBS associated with mock‐inoculated tissues are colored shades of purple. As in 6a and 6b, the TF and TFBS from *Psg*‐infected tissues are far more robust, confirming *Psg* infection enhances responses to *e*CO_2_.

### *e*CO_2_ suppresses viral immunity

**Fig. 7:**
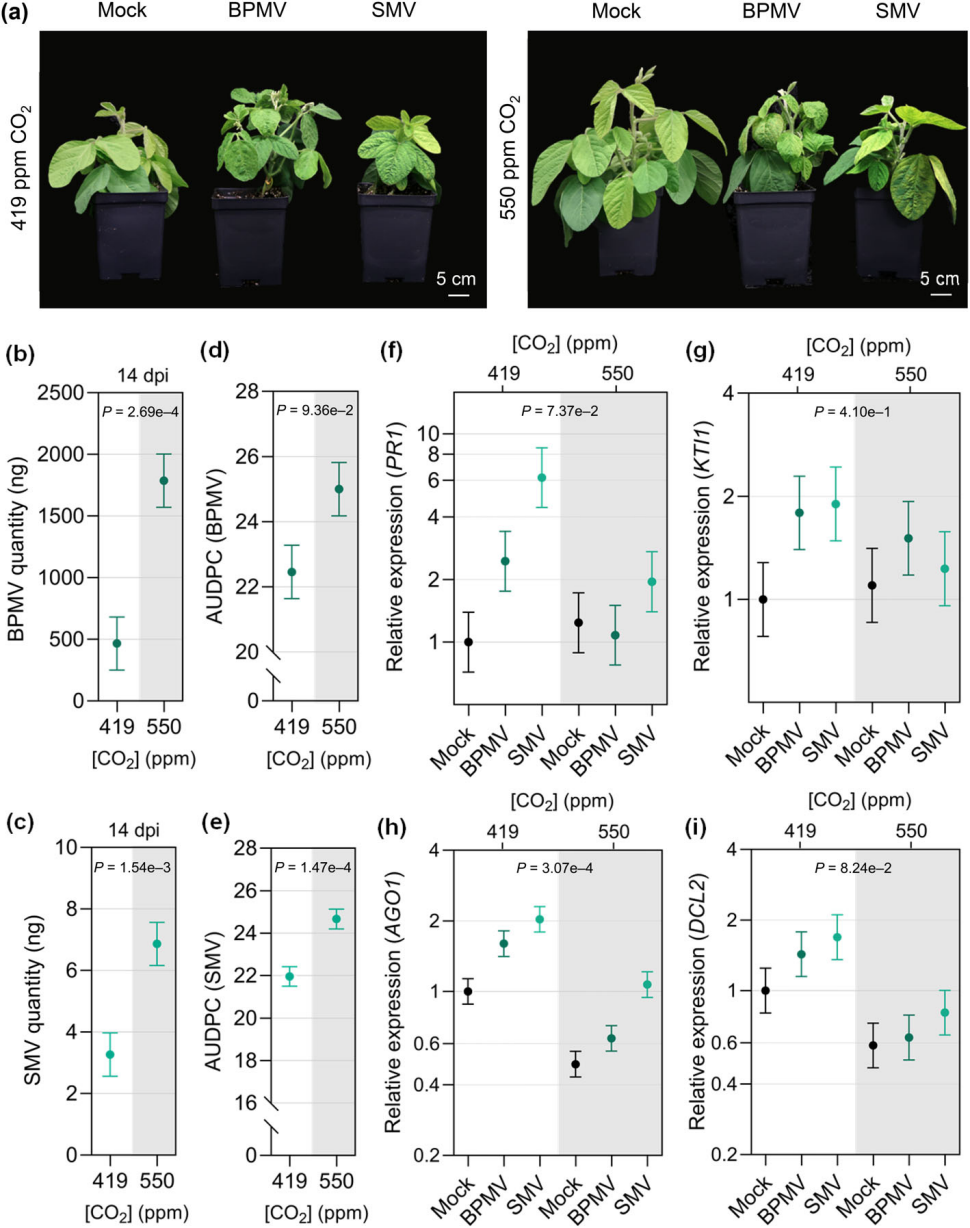
Young soybean plants growing in elevated CO_2_ (*e*CO_2_) are more susceptible to bean pod mottle virus (BPMV) and soybean mosaic virus (SMV). (a) Representative photos of soybean growth in response to *e*CO_2_ during infection with BPMV or SMV compared with mock‐treated control plants. Plants were photographed at 21 d post‐inoculation (dpi). (b) BPMV and (c) SMV quantity at 14 dpi was assessed by reverse transcriptase‐quantitative polymerase chain reaction using *Skp1* as a housekeeping gene. Disease progression of (d) BPMV and (e) SMV calculated as the area under the disease progress curve (AUDPC) over a 35‐d time course. Expression of (f) *PR1* (SA marker gene) (g) *KTI1* (JA marker gene), and RNA‐silencing‐related genes (h) *AGO1* and (i) *DCL2* in response to BPMV, SMV, or mock treatment was assessed by reverse transcription quantitative polymerase chain reaction analysis relative to the expression of *Skp1*. The newest fully expanded leaves of eight plants were sampled at the indicated time point for each treatment. The three experimental replicates were conducted simultaneously in independent CO_2_ controlled chambers using a replicated complete randomized design. Data were graphed as the mean across the three replicates with SE bars. *P*‐values were computed using *F*‐tests for the main effect of CO_2_ from the linear mixed effect model (LMM) analysis on the log‐transformed relative gene expression data. The following *P*‐values were associated with the log‐transformed relative *PR1* expression of virus‐infected plants compared to mock‐treated plants at ambient CO_2_ condition: p(BPMV_PR1,419_) = 0.1282, p(SMV_PR1,419_) = 0.0178 and at *e*CO_2_ condition: p(BPMV_PR1,550_) = 0.7779, p(SMV_PR1,550_) = 0.3882. All was calculated based on *t*‐tests for the contrasts between each pathogen and mock at the corresponding CO_2_ conditions from the LMM analysis. The following *P*‐values were computed using *t*‐tests from the LMM analysis for the interaction effect between CO_2_ condition and pathogen treatment (virus or mock) on log‐transformed *KTI1* gene expression: p(BPMV_KTI1_) = 0.6156 and p(SMV_KTI1_) = 0.3444. The letter e denotes an exponent to the power of 10.

To determine whether *e*CO_2_ affects virus susceptibility in soybean, we inoculated the unifoliate leaves of 14‐d‐old plants with two virus species, BPMV, a *Comovirus* in the family *Secoviridae* (Sanfaçon *et al*., 2009), and SMV, a *Potyvirus* in the family *Potyviridae* (Hajimorad *et al*., 2018). BPMV and SMV caused a greater reduction in soybean growth and shoot dry weight when compared to the mock plants in *e*CO_2_ than in *a*CO_2_ (Figs 7a, S6). Accumulation of BPMV and SMV was higher in the newest fully expanded trifoliate leaves of infected plants grown at *e*CO_2_ compared to those grown at *a*CO_2_ at 14 dpi (Fig. 7b,c) and to a lesser extent at 21 dpi (Fig. S7). Accordingly, the AUDPC was greater for BPMV‐ and SMV‐infected plants grown at *e*CO_2_ (Fig. 7d,e). We used reverse transcription quantitative polymerase chain reaction to assay expression of soybean homologs of four genes associated with defense responses to viruses: *PR1*, *KTI1*, *AGO1*, and *DCL2*. We found that the overall mean expression of the SA marker gene, *PR1*, was lower at *e*CO_2_ when considering both mock‐ and virus‐infected plants (Fig. 7f). In addition, expression of *PR1* was induced in both BPMV‐ and SMV‐infected plants relative to mock‐inoculation under *a*CO_2_. However, *PR1* expression in the virus‐infected plants was similar to the mock‐inoculated plants in *e*CO_2_. The expression of *KTI1* was similar under the two CO_2_ levels (Fig. 7g), and its induction in virus‐infected vs mock plants was not significantly different between *a*CO_2_ and *e*CO_2_, suggesting that JA‐mediated defenses against viruses were not affected. We also observed that *AGO1* and *DCL2* accumulated to lower levels in plants grown at *e*CO_2_ (Fig. 7h,i), suggesting that RNA silencing‐based defenses could potentially be compromised (Leonetti *et al*., 2021; Akbar *et al*., 2022). These data suggest that the increased susceptibility of soybean to BPMV and SMV under *e*CO_2_ may be partially mediated by suppression of SA‐related signaling and RNA‐silencing mechanisms.

### Susceptibility to soil‐borne filamentous pathogens is altered under elevated CO_2_

**Fig. 8:**
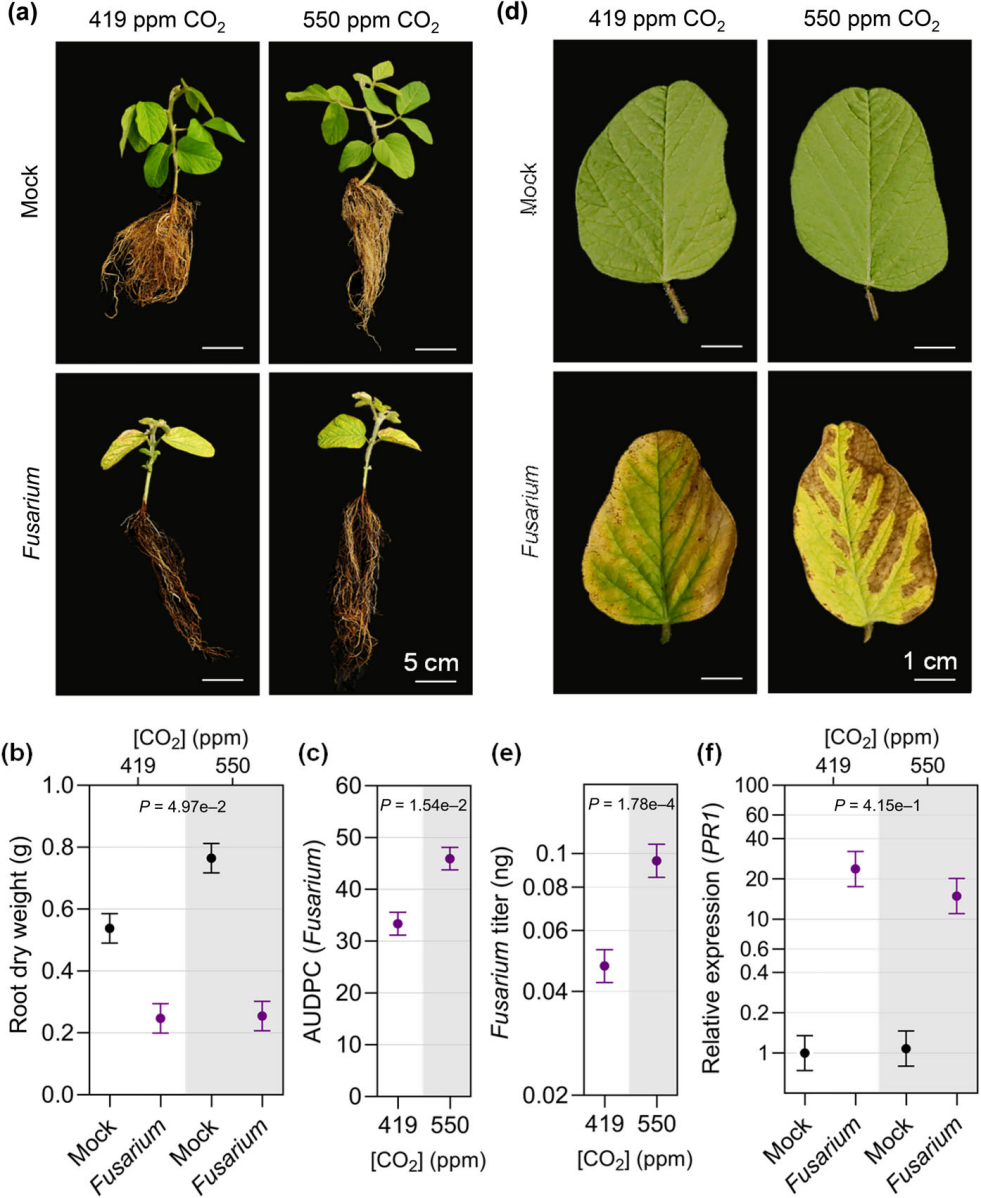
Young soybean plants growing in elevated CO_2_ (*e*CO_2_) develop more sudden death syndrome (SDS) symptoms and are more susceptible to *Fusarium virguliforme*. (a) Representative photos of soybean plants at 35 d after planting (dap) that were mock‐treated or germinated in soil infested with *F. virguliforme* (*Fusarium*) and (b) associated root dry weight. (c) SDS disease progression, assessed as area under the disease progress curve (AUDPC), and (d) close‐up photographs of SDS disease symptoms on unifoliate leaves at 35 dap. (e) *F. virguliforme* titer was assessed by quantitative polymerase chain reaction and (f) expression of *PR1* (SA marker gene) in soybean roots at 35 dap was assessed by reverse transcriptase‐quantitative polymerase chain reaction analysis relative to the *Skp1* housekeeping gene. The three experimental replicates were conducted simultaneously in independent CO_2_ controlled chambers using a replicated complete randomized design. Data were graphed as the mean across the three replicates with SE bars. *P*‐values were computed using *F*‐tests for the main effect of CO_2_ on AUDPC and log‐transformed titers, and interaction effect between CO_2_ and *Fusarium* treatment on root dry weight and log‐transformed *PR1* gene expression from the linear mixed effect model analysis. The letter e denotes an exponent to the power of 10.

To determine whether *e*CO_2_ also impacts susceptibility to soil‐borne pathogens, we inoculated soybean with *F. virguliforme*, a hemibiotrophic fungus causing SDS (O'Donnell *et al*., 2010), or *P. sylvaticum*, a necrotrophic oomycete causing seed decay and seedling root rot (Rojas *et al*., 2017). In mock‐inoculated plants, root dry weight was increased in *e*CO_2_ relative to *a*CO_2_ (*P* = 0.0098), which is consistent with the increased shoot dry weight and overall CO_2_ fertilization effect in soybean (Fig. 1). Root and shoot dry weight were decreased to a greater extent in *F. virguliforme‐*infected plants grown in *e*CO_2_ compared with plants grown under *a*CO_2_ at 35 dap (Figs 8a,b, S8). Correspondingly, progression of the foliar symptoms of SDS was greater (Fig. 8c) and more severe symptoms developed on the leaves under *e*CO_2_ (Fig. 8d). Consistent with the increased disease symptoms, *F. virguliforme* was more abundant in the tap roots of plants grown in *e*CO_2_ at 35 dap (Fig. 8e). To investigate whether expression of SA‐ or JA‐related defense genes was affected in *e*CO_2_, accumulation of *PR1* and *KTI1* mRNA transcripts, respectively, was quantified in soybean roots using reverse transcription quantitative polymerase chain reaction. Expression of neither gene was convincingly altered in response to *F. virguliforme* infection in *e*CO_2_ relative to plants grown under *a*CO_2_ (Figs 8f, S9). To investigate whether *F. virguliforme* growth is affected by [CO_2_], we measured the diameter of *F. virguliforme* colonies as they grew on plates for 11 d in *a*CO_2_ or *e*CO_2_. Fungal growth was similar at the two CO_2_ levels (Fig. S10), indicating that the greater fungal accumulation in roots and SDS progression observed in soybeans at *e*CO_2_ is not caused by increased pathogen replication rate. Together, our data suggest that *e*CO_2_ increases susceptibility to *F. virguliforme*, although the underlying molecular mechanisms mediating this altered interaction are unclear.

**Fig. 9:**
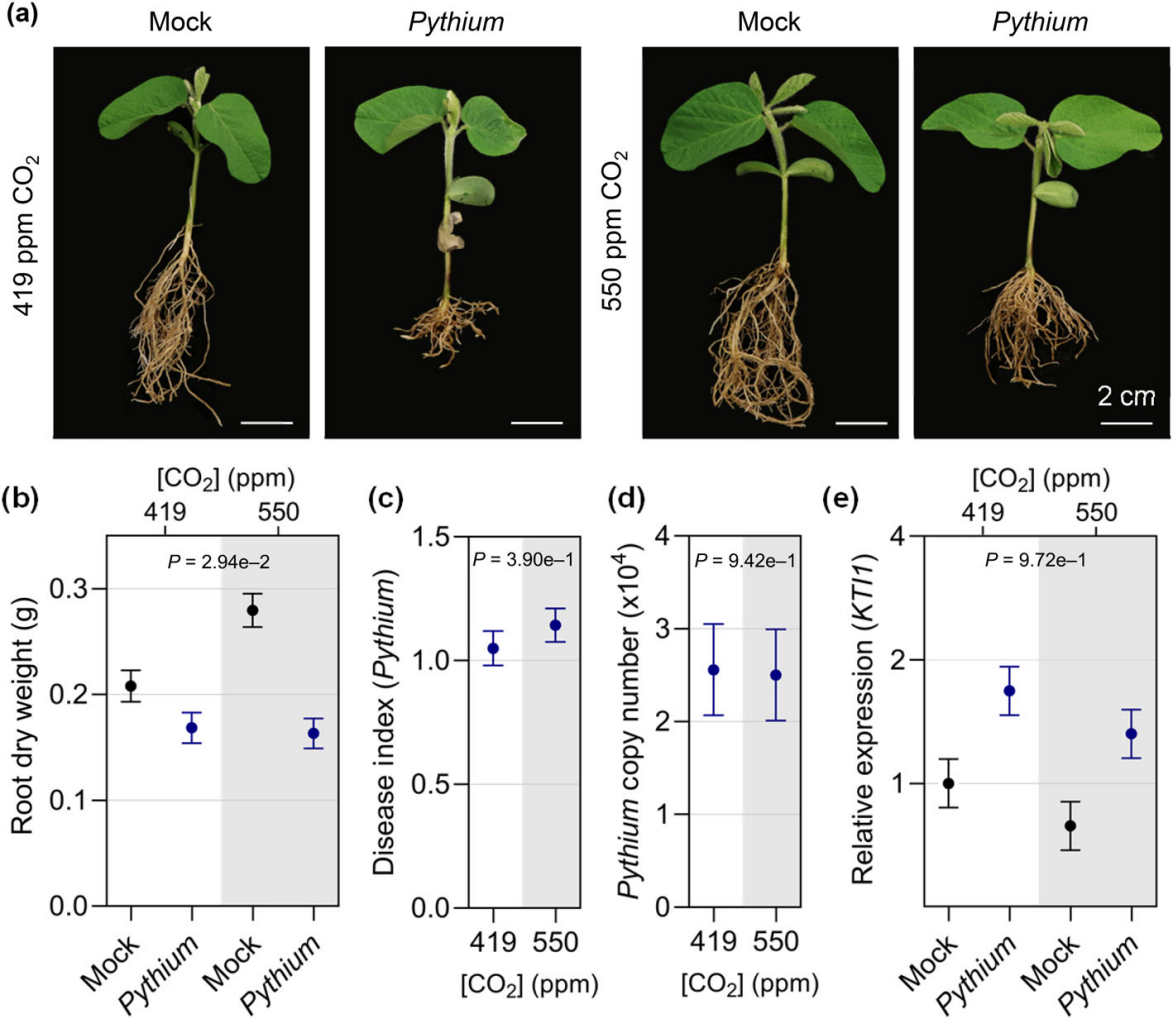
Effects of elevated CO_2_ (*e*CO_2_) on *Pythium sylvaticum* infection in soybean. (a) Representative photos of soybean plants at 21 d after planting (dap) that were mock‐treated or germinated in soil infested with *P. sylvaticum* (*Pythium*) and (b) associated root dry weight. (c) *P. sylvaticum* disease index measured at 14 dap and (d) *P. sylvaticum* copy number in roots determined by quantitative polymerase chain reaction analysis at 14 dap. (e) *KTI1* (JA marker gene) expression in response to mock treatment or *P. sylvaticum* infection assessed by reverse transcriptase‐quantitative polymerase chain reaction analysis. Gene expression was assessed relative to the *Skp1* housekeeping gene. The three experimental replicates were conducted simultaneously in independent CO_2_‐controlled chambers using a replicated complete randomized design. Data were graphed as the mean across the three replicates with SE bars. *P*‐values were computed using *F*‐tests for the main effect of CO_2_ on area under disease progress curve and *Pythium* copy number, and interaction effect between CO_2_ and *Pythium* treatment on root dry weight and log‐transformed *KTI1* expression from the linear mixed effect model analysis. The letter e denotes an exponent to the power of 10.

*Pythium sylvaticum* caused noticeable root rot under both CO_2_ concentrations (Fig. 9a). Although mock‐inoculated plants exhibited an increase in root dry weight at *e*CO_2_ (*P*‐value = 0.0092), the root and shoot dry weights of *P. sylvaticum*‐infected plants were more dramatically decreased compared with the mock treatment under *e*CO_2_ relative to *a*CO_2_ (Figs 9b, S11). Hyphal growth assays indicated that *P. sylvaticum* grew more quickly in *e*CO_2_ than in *a*CO_2_ (Fig. S12). However, disease index ratings were similar between infected plants grown under both [CO_2_] (Fig. 9c), which was consistent with the similar *P. sylvaticum* titers observed in roots at 14 dap (Fig. 9d). Additionally, no difference was observed in *KTI1* expression (Fig. 9e), and while the *P*‐value for *PR1* expression was below 0.05, we do not consider it to be significant because of the similar levels of expression in *Pythium*‐infected tissue (Fig. S13) at 14 dap between the two [CO_2_]. Together, our results suggest that *e*CO_2_ could have a minor effect on *in vitro* growth of *P. sylvaticum*; however, we could not demonstrate that this led to the pathogen being more aggressive in the roots. It is also interesting that while susceptibility to *P. sylvaticum* appears to be unchanged, there is a greater loss in root and shoot biomass in plants grown in *e*CO_2_.

## Discussion

Given the central role of CO_2_ in plant biology and its increasing abundance in the atmosphere, there are many effects on plant physiology that can potentially impact interactions with pathogenic microbes (Bazinet *et al*., 2022). While some themes are beginning to emerge, it remains challenging to predict the response of a given plant to a given pathogen under *e*CO_2_. Here, we selected a diverse panel of phytopathogens to assess whether and how soybean–microbe interactions are impacted by *e*CO_2_. Our results demonstrate that CO_2_ levels expected by mid‐century (Jaggard *et al*., 2010) have significant impacts on soybean immunity that varied across the different pathogen types.

Extensive research from SoyFACE experiments has demonstrated that under favorable environmental conditions, *e*CO_2_ (550 ppm CO_2_) has the potential to benefit soybean growth and yield (Long *et al*., 2006; Ainsworth & Long, 2021). In line with field results, we observed that leaf photosynthetic rate and plant biomass increased, whereas stomatal conductance, density, and aperture decreased (Fig. 1). Interestingly, we found that expression of some photosynthesis‐related genes, such as *Rubisco small subunit 1B* (*RbcS1B*), decreased under eCO_2_, which seems contradictory to the enhanced photosynthesis. However, others have shown that mRNA expression of *RbcS* and other photosynthesis‐related genes can be downregulated as nonstructural sugars accumulate and plants begin to acclimate to *e*CO_2_ (Cheng *et al*., 1998; Thompson *et al*., 2017). The combined results indicated that the soybean plants were performing as expected in *e*CO_2_ under our growth conditions.

The impacts of *e*CO_2_ on disease are limited to one study that investigated the incidence and severity of three naturally occurring diseases over 3 years in SoyFACE (Eastburn *et al*., 2010). *e*CO_2_ did not affect SDS incidence or severity, but the severity of brown spot disease (*Septoria glycines*) increased, while the severity of downy mildew (*Peronospora manshurica*) decreased. The results from this study are consistent with the idea that diseases caused by necrotrophic pathogens may be typically enhanced under *e*CO_2_, whereas diseases caused by biotrophic and hemibiotrophic pathogens may be suppressed in C_3_ plants (Bazinet *et al*., 2022). Our goal was to extend these findings under controlled environmental conditions to determine how *e*CO_2_ differentially affects responses to distinct types of pathogens and to establish a foundation to understand the complex and dynamic signaling events that influence soybean–pathogen interactions in response to *e*CO_2_.

The effects of [CO_2_] on bacterial infection have not been investigated previously in soybean; however, several independent studies have indicated that for the hemibiotrophic pathogen *Pst*DC3000, *e*CO_2_ enhances resistance in tomato (Li *et al*., 2015; Zhang *et al*., 2015; Hu *et al*., 2021) and mostly increases susceptibility in Arabidopsis (Zhou *et al*., 2017, 2019). Arabidopsis does not become more susceptible to all pathogens, as it is more resistant to infection with the necrotrophic pathogen *B. cinerea* at *e*CO_2_ (Zhou *et al*., 2019). In our work, we observed enhanced resistance in compatible interactions between *Psg* and soybean under *e*CO_2_, and to a lesser extent, during incompatible interactions with *Pst*DC3000 and *Pst*DC3000 *hrcC‐* (Fig. 3a). Taken together, it is evident that bacterial immunity is altered under *e*CO_2_ in C3 plants in a pathosystem‐specific manner.

Phytohormones and stomatal immunity play pivotal roles in altering defense responses to bacteria under *e*CO_2_ in Arabidopsis and tomato. For example, in tomato, enhanced resistance to *Pst*DC3000 was associated with *e*CO_2_‐induced stomatal closure (Li *et al*., 2015) and higher SA biosynthesis (Li *et al*., 2015; Zhang *et al*., 2015). In Arabidopsis, increased susceptibility to *Pst*DC3000 was associated with reduced abscisic acid (ABA) levels, causing changes in stomatal dynamics favoring bacterial infection (Zhou *et al*., 2017), and shifts in SA/JA antagonism toward JA‐mediated immunity against necrotrophic pathogens (Zhou *et al*., 2019). In response to *Psg* infection, we observed a 1.7‐fold increase in SA accumulation and no notable change in JA levels (Fig. S4). However, this slight increase in SA seems unlikely to provide the level of resistance against *Psg* observed under *e*CO_2_ based on previous work showing that free SA was increased by seven‐ to tenfold in soybean plants exhibiting SA‐dependent, constitutive defense responses (Liu *et al*., 2011). We hypothesize that enhanced bacterial resistance is likely conferred by a combination of physiological, metabolic, and molecular responses that together limit bacterial infection under *e*CO_2_. Indeed, we observed an upregulation of MAMP‐triggered immune responses in soybeans at *e*CO_2_ including flg22‐induced oxidative species production (Fig. 4b) and MAPK activation (Fig. 4c). We additionally observed reduced stomatal density (Fig. 1c) and a constitutive decrease in stomatal aperture (Fig. 1d), which is likely to limit the entry of bacterial species.

Our transcriptomic analysis also indicates that *e*CO_2_ has direct impacts on soybean defense. *e*CO_2_ caused an overrepresentation of DEGs associated with defense responses (Fig. 2; Table S5), even in the absence of pathogen challenge. Most notable was altered expression of genes associated with JA signaling and response to JA, including decreased expression of *Jasmonate Zim Domain* (*JAZ*)‐encoding genes, which repress jasmonate signaling, and *MYC2* orthologs, known as the master regulators of jasmonate signaling (Johnson *et al*., 2023). Although we did not observe higher JA levels under *e*CO_2_ in our metabolite analysis (Fig. S4), downregulation of these genes suggests that JA‐mediated responses may be altered in soybean plants growing in *e*CO_2_. This finding is consistent with prior work demonstrating that various insects prefer to feed on and cause more damage to plants grown in *e*CO_2_ (DeLucia *et al*., 2008; Johnson *et al*., 2020), which has been attributed at least in part to compromised JA‐mediated defenses (Zavala *et al*., 2008).

While we focused on JA in our studies, we are aware that ethylene often works cooperatively with JA in plant defense, and there is also evidence that ethylene biosynthesis and response may be affected in soybean in *e*CO_2_. Casteel *et al*. (2008) found that mRNA transcripts for a gene encoding 1‐aminocyclopropane 1‐carboxylate (ACC) synthase were downregulated in *e*CO_2_ in SoyFACE before and after beetle damage. However, neither ethylene biosynthesis nor response were enriched GO terms in our experiments. In soybean, there are at least 21 genes annotated as ACC synthase and 16 annotated as ACC oxidase, which catalyze the final steps in ethylene biosynthesis (Arraes *et al*., 2015), but none were differentially expressed in the newest fully expanded trifoliate leaves at 21 dap (Table S4). However, many of these genes were differentially expressed in the unifoliate leaves following *Psg* inoculation in both CO_2_ treatments (Table S6), and of these, there were no ACC synthase genes that were differentially expressed when comparing response to *Psg* in *e*CO_2_ vs *a*CO_2_ (Table S8). There were six ACC oxidase genes differentially regulated in response to *Psg* in *e*CO_2_ vs *a*CO_2_ (Table S8), and five of these have higher expression in *e*CO_2_. These observations suggest that ethylene biosynthesis and response were not downregulated in our experiments and warrant further investigations into the roles of ethylene in soybean–pathogen interactions in future climate scenarios.

We also found no evidence for constitutive upregulation of SA‐mediated defenses under *e*CO_2_. However, some genes that positively regulate basal defense responses were upregulated, suggesting that some immune responses might be primed in soybean under these conditions. The effects of *e*CO_2_ on DEGs were even more pronounced following *Psg* infection (Fig. 5; Table S8). Similar DEGs were identified at *a*CO_2_ and *e*CO_2_ in response to *Psg* infection; however, the amplitude of these responses were significantly more up‐ or downregulated, depending on the gene, in response to *e*CO_2_ (Table S8). This is in line with the higher *Psg*‐ and flg22‐elicited defense gene expression and basal defense responses under *e*CO_2_.

The effects of *e*CO_2_ on virus susceptibility have been studied in various other C3 plants, most of which have reported increased resistance to viral pathogens associated with a constitutive overproduction of SA (Fu *et al*., 2010; Trębicki *et al*., 2016) or upregulation of RNA silencing genes that are critical for viral defense (Guo *et al*., 2020). As discussed previously, we did not observe a constitutive stimulation of SA levels or SA‐based defenses in our experimental system, and interestingly, we found that expression of two genes potentially involved in antiviral RNA silencing were decreased (Fig. 7h,i). In line with our molecular analyses, soybean plants were more susceptible to BPMV and SMV in *e*CO_2_. Increased susceptibility could be partially explained by reduced induction of SA‐based defenses, as indicated by reduced *PR1* gene expression in virus‐treated plants in *e*CO_2_ (Fig. 7f). It is also possible that changes in RNA‐silencing components are responsible for increased susceptibility, as we observed a reduction in both *AGO1* (Fig. 7h) and *DCL2* (Fig. 7i) expression in virus‐infected soybean at *e*CO_2_. An interesting avenue to pursue in the future would be to test whether there is a link between the apparent downregulation of SA‐mediated defenses and antiviral RNA silencing.

Many viruses, including BPMV and SMV, are transmitted by an insect vector in nature. Importantly, many of the studies indicating that plants are less susceptible to viruses under *e*CO_2_ have used aphids as a vector for viral delivery. Thus, we do not know whether the presence of bean leaf beetle (BPMV) or aphid (SMV) may influence the outcome of the interaction. Furthermore, other factors may be influenced by *e*CO_2_ in ways that could affect dissemination of the viruses. For example, the larger canopy size associated with increased photosynthetic rates in soybean are expected to cause a higher dispersion rate, increasing disease incidence, and severity in the field (Amari *et al*., 2021). It will be interesting to study the tri‐trophic interactions in the future from mechanistic and epidemiological perspectives, and perhaps including other climate change‐associated gasses such as ozone, which retards SMV infection in soybean (Bilgin *et al*., 2008).

*e*CO_2_ has been shown to have various effects on diseases caused by fungi and oomycetes (filamentous pathogens). While various studies have investigated the effect of *e*CO_2_ on filamentous pathogens (Smith & Luna, 2023), relatively few have examined soil‐borne pathogens. Elevated CO_2_ (800 ppm) had no effect on infections caused by *Rhizoctonia solani* or *F. oxysporum* in Arabidopsis plants (Zhou *et al*., 2019). Tomato plants grown in 700 ppm CO_2_ were more tolerant to root rot caused by *Phytophthora infestans* (Jwa & Walling, 2001). Growth of *P. infestans* was not directly affected by [CO_2_] and the increased resistance of tomato plants could not be associated with changes in expression of marker genes associated with SA‐, ABA‐, or JA‐based defenses. In FACE experiments involving natural infections, *e*CO_2_ led to higher incidence of sheath blight (*Rhizoctonia solani*) on rice (Kobayashi *et al*., 2006), but SDS (*F. virguliforme*) in soybean was not affected (Eastburn *et al*., 2010). Interestingly, the increased incidence of rice sheath blight was only observed in plots that also received input of high nitrogen fertilizer.

Our data indicate that soybean plants are more susceptible to *F. virguliforme* under *e*CO_2_ but had no notable change in *P. sylvaticum* infection. Surprisingly, we observed a greater loss in root and shoot dry weight in response to *P. sylvaticum* even though visual disease scores were similar, and similar quantities of *P. sylvaticum* DNA were observed in the roots of plants grown at *e*CO_2_ and *a*CO_2_. These observations suggest that the CO_2_ fertilization effect was lost due to *P. sylvaticum* infection. We only observed a modest reduction in *PR1* expression following *F. virguliforme* treatment under *e*CO_2_, and there was no change in expression of the *KTI1* JA marker gene. These gene expression results are similar to what has previously been observed in tomato with increased susceptibility showing no correlation with concomitant changes in expression of defense pathway marker genes (Jwa & Walling, 2001). Various other factors have been linked to *F. virguliforme* resistance, which we did not assess in this study, including the production of phytoalexins and changes in root exudates, both of which can be affected by *e*CO_2_ (Vaughan *et al*., 2014; Usyskin‐Tonne *et al*., 2020). Experiments at FACE facilities have demonstrated dramatic changes in the soil microbial rhizosphere and endosphere (Gao *et al*., 2022; Rosado‐Porto *et al*., 2022), which could also impact the composition of phytopathogenic fungi in the surrounding soil and cause changes in soybean–pathogen interactions. One could also expect that changes in root architecture and growth could benefit or curtail infection by soil pathogens. Our data indicate that disease losses due to soil‐borne filamentous pathogens can be exacerbated in soybean grown in *e*CO_2_, and there is much more to learn about the underlying mechanisms.

In conclusion, we have demonstrated that soybean basal defense responses and gene expression are altered under *e*CO_2_, differentially regulating susceptibility to bacterial, viral, fungal, and oomycete pathogens. In our experiments, we assessed the effect of [CO_2_] on pathogen growth, however, we did not investigate how pathogen virulence or evolution affects soybean–pathogen interactions under these conditions. In our study, we focused on the impact of one factor, *e*CO_2_, on soybean physiology and disease susceptibility. However, *e*CO_2_ is not the only environmental factor to consider when predicting the outcome of future climatic conditions on plant health. Increases in temperature, changes in water and nutrient availability, soil pH, photoperiod, and severe weather events are also expected to affect disease susceptibility (Saijo & Loo, 2020). Understanding how combined stresses, in the context of future atmospheric CO_2_ levels that are likely to occur, affect defense signaling and disease development is a critical question that needs to be investigated further to develop new strategies to mitigate crop losses due to threats imposed by climate change.

## Competing interests

None declared.

## Author contributions

EK, MB, ASC and KLH performed the experiments. MB, EK and SAW conceived of the experiments. MWB performed the metabolite analysis. YQ and PL performed statistical analysis using the linear mixed models. MAG performed the QuantSeq 3′ mRNA‐Seq data analysis. MB, EK, MAG and SAW wrote the manuscript with contributions from all authors. MB, EK and ASC contributed equally to this work.

## Disclaimer

The New Phytologist Foundation remains neutral with regard to jurisdictional claims in maps and in any institutional affiliations.

## Supporting information


**Fig. S1** Schematic representation of growth chamber conditions.
**Fig. S2** Stomatal index on the abaxial leaf surface of soybean.
**Fig. S3** Effect of elevated CO_2_ on growth rate of *Pseudomonas syringae* pv. *tomato* DC3000 (*Pst*DC3000).
**Fig. S4** Accumulation of salicylic acid and jasmonic acid at 6 h post inoculation with *Pseudomonas syringae* pv. *glycinea* in ambient CO_2_ and elevated CO_2_.
**Fig. S5** Hierarchical clustering of 21 822 differentially expressed genes (FDR < 0.01) responding to *Pseudomonas syringae* pv. *glycinea* at 6 h post inoculation in plants grown in elevated CO_2_ (550 parts per million (ppm)) or ambient CO_2_ (419 ppm) conditions.
**Fig. S6** Effect of elevated CO_2_ on the shoot fresh weight and shoot dry weight of soybean plants infected with bean pod mottle virus and soybean mosaic virus.
**Fig. S7** Bean pod mottle virus and soybean mosaic virus accumulation at 21 d post inoculation in soybean plants growing in ambient CO_2_ and elevated CO_2_.
**Fig. S8** Effects of elevated CO_2_ on root and shoot biomass of soybean plants infected with *Fusarium virguliforme*.
**Fig. S9** Expression of the jasmonic acid marker gene, *KTI1*, in roots of plants infected with *Fusarium virguliforme* in ambient CO_2_ and elevated CO_2_.
**Fig. S10** CO_2_ concentration does not impact *in vitro* growth of *Fusarium virguliforme*.
**Fig. S11** Soybean root and shoot biomass is impacted by elevated CO_2_ in *Pythium sylvaticum‐*infected plants.
**Fig. S12** *In vitro* growth of *Pythium sylvaticum* is accelerated under elevated CO_2_.
**Fig. S13** Expression of the salicylic acid marker gene, *PR1*, in roots of plants infected with *Pythium sylvaticum* in ambient CO_2_ and elevated CO_2_.
**Methods S1** QuantSeq 3′mRNA‐Seq data analysis and JA and SA quantification.
**Table S1** List of primers and probes used in this study.
**Table S2** Sudden death syndrome rating scale of foliar symptoms ranging from 0 to 7.
**Table S3** Disease rating scale of *Pythium sylvaticum* root symptoms.

**Table S4** Significantly differentially expressed genes (FDR < 0.01) identified by comparing Williams 82 grown at elevated CO_2_ (550 parts per million (ppm)) vs ambient CO_2_ (419 ppm) conditions for 21 d.
**Table S5** Gene Ontology biological process terms significantly enriched in the STRING (v12) network developed from clusters of differentially expressed genes responding to elevated (550 parts per million (ppm)) vs ambient CO_2_ conditions (419 ppm) for 21 d.
**Table S6** Significantly differentially expressed genes (FDR < 0.01) responding to *Pseudomonas syringae* pv. *glycinea* infection (6 h) in plants grown in elevated CO_2_ (550 parts per million (ppm)) or ambient CO_2_ (419 ppm) conditions.
**Table S7** Gene Ontology biological process terms significantly overrepresented among differentially expressed gene clusters comparing *Pseudomonas syringae* pv. *glycinea* response (*Psg* v. Mock) in elevated CO_2_ (550 parts per million (ppm)) and/or ambient CO_2_ (419 ppm) CO_2_ conditions (419 ppm).
**Table S8** Significantly differentially expressed genes (FDR < 0.01) responding to changes in CO_2_ (elevated (*e*CO_2_) 550 parts per million (ppm) or ambient (*a*CO_2_) 419 ppm) in *Pseudomonas syringae* pv. *glycinea*‐infected (6 h) and/or mock‐infected leaves.
**Table S9** Gene Ontology biological process terms significantly enriched in the STRING (v12) network developed from clusters of differentially expressed genes responding to elevated (550 parts per million (ppm)) vs ambient CO_2_ conditions (419 ppm) in *Pseudomonas syringae* pv. *glycinea* and/or mock‐infected leaves at 21 d.
**Table S10** Annotation of significantly differentially expressed transcription factors (FDR < 0.01) responding to changes in CO_2_ (elevated (*e*CO_2_) 550 parts per million (ppm) or ambient CO_2_ 419 ppm) in *Pseudomonas syringae* pv. *glycinea*‐infected (6 h) and/or mock‐infected leaves.
**Table S11** Identification of transcription factors with overrepresented binding sites among the promoters of differentially expressed genes responding to changes in CO_2_ (elevated (*e*CO_2_) 550 parts per million (ppm), ambient CO_2_ 419 ppm) in *Pseudomonas syringae* pv. *glycinea*‐infected (6 h) and/or mock‐infected leaves.
**Table S12** Differentially expressed gene targets of transcription factors with overrepresented binding sites (Table S10) responding to changes in CO_2_ (elevated (*e*CO_2_) 550 parts per million (ppm), ambient CO_2_ 419 ppm) in *Pseudomonas syringae* pv. *glycinea*‐infected (6 h) and/or mock‐infected leaves.
**Table S13** Gene Ontology biological process terms significantly overrepresented among differentially expressed gene targets of transcription factors with overrepresented transcription factor binding sites in expression clusters from *Pseudomonas syringae* pv. *glycinea* and/or mock‐infected leaves responding to CO_2_ levels (elevated = 550 parts per million (ppm) vs ambient = 419 ppm).
Please note: Wiley is not responsible for the content or functionality of any Supporting Information supplied by the authors. Any queries (other than missing material) should be directed to the *New Phytologist* Central Office.

## Data Availability

The RNA‐seq reads for QuantSeq dataset 1 and dataset 2 were deposited in NCBI under BioProject PRJNA1017882 and PRJNA1017884, respectively. The raw data and statistical reports from R that underlie the graphs in Figs 1, 3, 4, and 7, 8, 9 and Figs S2–S4 and S6–S13 were deposited at the Iowa State University DataShare, and they can be accessed using this Doi: 10.25380/iastate.27287847. The authors declare that any additional supporting data for this work can be found in the manuscript and its Supporting Information.
