# Supplementary material for: Elevated CO2 alters soybean physiology and defense responses, and has disparate effects on susceptibility to diverse microbial pathogens
Source: New Phytol. 2025 Jan 9;246(6):2718–37. doi: 10.1111/nph.20364 (PMC12095978; doi:10.1111/nph.20364)
Supplement: Supplementary file 1 — Fig. S1 Schematic representation of growth chamber conditions. Fig. S2 Stomatal index on the abaxial leaf surface of soybean. Fig. S3 Effect of elevated CO2 on growth rate of Pseudomonas syringae pv. tomato DC3000 (PstDC3000). Fig. S4 Accumulation of salicylic acid and jasmonic acid at 6 h post inoculation with Pseudomonas syringae pv. glycinea in ambient CO2 and elevated CO2. Fig. S5 Hierarchical clustering of 21 822 differentially expressed genes (FDR < 0.01) responding to Pseudomonas syringae pv. glycinea at 6 h post inoculation in plants grown in elevated CO2 (550 parts per million (ppm)) or ambient CO2 (419 ppm) conditions. Fig. S6 Effect of elevated CO2 on the shoot fresh weight and shoot dry weight of soybean plants infected with bean pod mottle virus and soybean mosaic virus. Fig. S7 Bean pod mottle virus and soybean mosaic virus accumulation at 21 d post inoculation in soybean plants growing in ambient CO2 and elevated CO2. Fig. S8 Effects of elevated CO2 on root and shoot biomass of soybean plants infected with Fusarium virguliforme. Fig. S9 Expression of the jasmonic acid marker gene, KTI1, in roots of plants infected with Fusarium virguliforme in ambient CO2 and elevated CO2. Fig. S10 CO2 concentration does not impact in vitro growth of Fusarium virguliforme. Fig. S11 Soybean root and shoot biomass is impacted by elevated CO2 in Pythium sylvaticum‐infected plants. Fig. S12 In vitro growth of Pythium sylvaticum is accelerated under elevated CO2. Fig. S13 Expression of the salicylic acid marker gene, PR1, in roots of plants infected with Pythium sylvaticum in ambient CO2 and elevated CO2. Methods S1 QuantSeq 3′mRNA‐Seq data analysis and JA and SA quantification. Table S1 List of primers and probes used in this study. Table S2 Sudden death syndrome rating scale of foliar symptoms ranging from 0 to 7. Table S3 Disease rating scale of Pythium sylvaticum root symptoms. [file NPH-246-2718-s001.pdf]

New Phytologist

New Phytologist Supporting Information

Article title: Elevated CO_2 alters soybean physiology and defense responses, and has disparate effects on susceptibility to diverse microbial pathogens
Authors: Melissa Bredow, Ekkachai Khwanbua, Aline Sartor Chicowski, Yunhui Qi, Matthew W. Breitzman, Katerina L. Holan, Peng Liu, Michelle A. Graham, and Steven A. Whitham
Article acceptance date: 03 December 2024

The following Supporting Information is available for this article:

Fig. S1 Schematic representation of growth chamber conditions.

Fig. S2 Stomatal index on the abaxial leaf surface of soybean.

Fig. S3 Effect of elevated CO_2 on growth rate of *Pseudomonas syringae* pv. *tomato* DC3000 (*Pst*DC3000).

Fig. S4 Accumulation of SA and JA at 6 h post-inoculation with *Pseudomonas syringae* pv. *glycinea* (*Psg*) in *a*CO_2 and *e*CO_2.

Fig. S5 Hierarchical clustering of 21,822 DEGs (FDR < 0.01) responding to *Pseudomonas syringae* pv. *glycinea* (*Psg*) at 6 h post-inoculation in plants grown in *e*CO_2 (550 ppm) or *a*CO_2 (419 ppm) conditions.

Fig. S6 Effect of elevated CO_2 on the shoot fresh weight and shoot dry weight of soybean plants infected with bean pod mottle virus (BPMV) and soybean mosaic virus (SMV).

Fig. S7 Bean pod mottle virus (BPMV) and soybean mosaic virus (SMV) accumulation at 21 days post-inoculation in soybean plants growing in *a*CO_2 and *e*CO_2.

Fig. S8 Effects of *e*CO_2 on root and shoot biomass of soybean plants infected with *Fusarium virguliforme*.

Fig. S9 Expression of the JA marker gene, *KTI1*, in roots of plants infected with *Fusarium virguliforme* in *a*CO_2 and *e*CO_2.

Fig. S10 CO_2 concentration does not impact *in vitro* growth of *Fusarium virguliforme*.

Fig. S11 Soybean root and shoot biomass is impacted by *e*CO_2 in *Pythium sylvaticum* infected plants.

Fig. S12 *In vitro* growth of *Pythium sylvaticum* is accelerated under *e*CO_2.

Fig. S13 Expression of the SA marker gene, *PR1*, in roots of plants infected with *Pythium sylvaticum* in *a*CO_2 and *e*CO_2.

New Phytologist

Table S1 List of primers and probes used in this study.

Table S2 SDS rating scale of foliar symptoms ranging from 0 to 7.

Table S3 Disease rating scale of *P. sylvaticum* root symptoms.

Table S4 Significantly differentially expressed genes (DEGs, FDR < 0.01) identified by comparing Williams 82 grown at elevated CO_2 (*e*CO_2, 550 ppm) versus ambient CO_2 (*a*CO_2, 419 ppm) conditions for 21 days.

Table S5 Gene ontology (GO) biological process (BP) terms significantly enriched in the STRING (v12) network developed from clusters of differentially expressed genes (DEG) responding to elevated (550 ppm) versus ambient CO_2 conditions (419 ppm) for 21 days.

Table S6 Significantly differentially expressed genes (DEGs, FDR < 0.01) responding to *P. syringae* pv. *glycinea* (*Psg*) infection (6 hours) in plants grown in elevated (*e*CO_2, 550 ppm) or ambient CO_2 (*a*CO_2, 419 ppm) conditions.

Table S7 Gene ontology (GO) biological process (BP) terms significantly overrepresented among differentially expressed gene clusters comparing *P. syringae* pv. *glycinea* (*Psg*) response (*Psg* v. Mock) in elevated (*e*CO_2, 550 ppm) and/or ambient (*a*CO_2, 419 ppm) CO_2 conditions (419 ppm).

Table S8 Significantly differentially expressed genes (DEGs, FDR < 0.01) responding to changes in CO_2 (elevated (*e*CO_2) 550 ppm or ambient (*a*CO_2) 419 ppm) in *P. syringae* pv. *glycinea* (*Psg*)-infected (6 hours) and/or mock-infected leaves.

Table S9 Gene ontology (GO) biological process (BP) terms significantly enriched in the STRING (v12) network developed from clusters of DEGs responding to elevated (550 ppm) versus ambient CO_2 conditions (419 ppm) in *P. syringae* pv. *glycinea* (*Psg*) and/or mock-infected leaves at 21 days.

Table S10 Annotation of significantly differentially expressed transcription factors (TFs, FDR < 0.01) responding to changes in CO_2 (elevated (*e*CO_2) 550 ppm or ambient (*a*CO_2) 419 ppm) in *P. syringae* pv. *glycinea* (*Psg*)-infected (6 hours) and/or mock-infected leaves.

Table S11 Identification of transcription factors (TF) with overrepresented binding sites (TFBS) among the promoters of differentially expressed genes (DEG) responding to changes in CO_2 (elevated (*e*CO_2) 550 ppm, ambient (*a*CO_2) 419 ppm) in *P. syringae* pv. *glycinea* (*Psg*)-infected (6 hours) and/or mock-infected leaves.

Table S12 Differentially expressed gene (DEG) targets of transcription factors with overrepresented binding sites (Supplemental Table S10) responding to changes in CO_2 (elevated (*e*CO_2) 550 ppm, ambient (*a*CO_2) 419 ppm) in *P. syringae* pv. *glycinea* (*Psg*)-infected (6 hours) and/or mock-infected leaves.

Table S13 Gene ontology (GO) biological process (BP) terms significantly overrepresented

among differentially expressed gene (DEG) targets of transcription factors (TFs) with overrepresented transcription factor binding sites (TFBS) in expression clusters from *P. syringae* pv. *glycinea* (*Psg*) and/or mock-infected leaves responding to CO_2 levels (elevated=550 ppm versus ambient=419 ppm).

Methods S1 QuantSeq 3'mRNA-Seq data analysis and jasmonic acid (JA) and salicylic acid (SA) quantification.

Fig. S1 Schematic representation of growth chamber conditions.

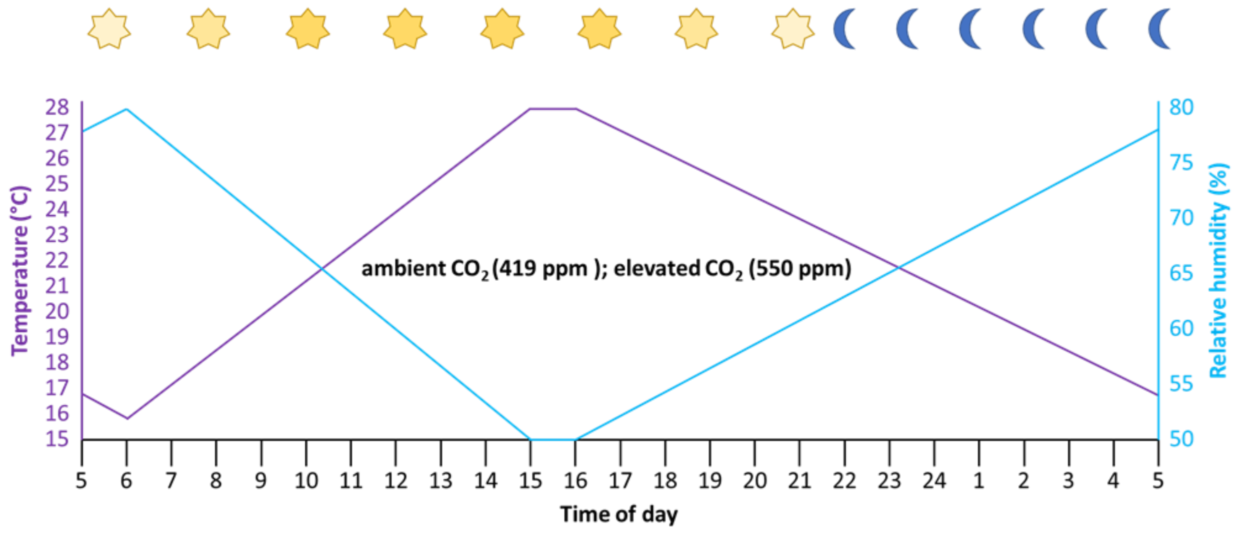

Fig. S1. Schematic representation of growth chamber conditions. Schematic representation of Enviratron growth chamber conditions. All chambers were set to the same conditions, except that CO_2 was 419 ppm in chambers set to ambient (*a*CO_2) conditions and CO_2 was 550 ppm in elevated (*e*CO_2) conditions. Lights were on at 5:38 am (05:38) and off at 8:52 pm (20:52) (sunrise and sunset times represent June 15 in central Iowa), minimum temperature was 15.6 °C at 6 am (06:00) with ramping to 27.6 °C maximum temperature from 3:00 – 4:00 pm (15:00 – 16:00). The relative humidity was ramped between 80% at 6:00 am (0:600) to the minimum of 50% at 3:00 – 4:00 pm (15:00-16:00).

Fig. S2 Stomatal index on the abaxial leaf surface of soybean.

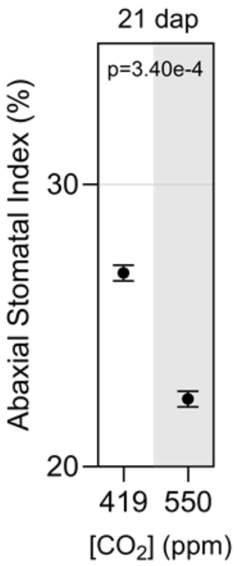

Fig. S2. Stomatal index on the abaxial leaf surface of soybean. Stomatal index measurements were performed as described in materials and methods. Three replicates were carried out simultaneously in independent CO_2 control chambers, using 5 plants per treatment for each CO_2 concentration. Stomata were counted in three randomly selected fields of view per leaf. Data points represent the mean value across the three replicates, and error bars represent the standard error. P-value was computed based on an F-test for the main effect of CO_2 in linear mixed effect model analysis. The letter e denotes an exponent to the power of 10, and dap indicates days after planting.

Fig. S3 Effect of elevated CO_2 on growth rate of *Pseudomonas syringae* pv. *tomato* DC3000 (*Pst*DC3000).

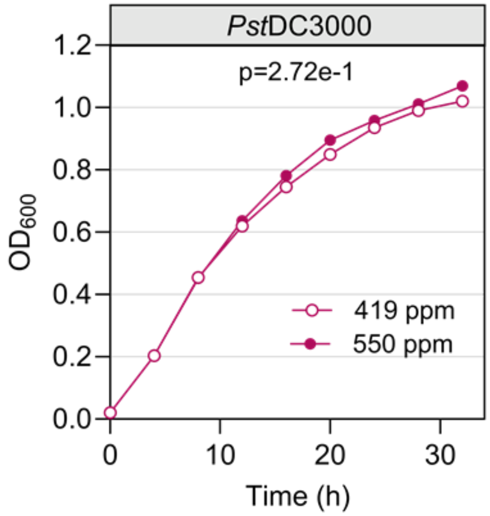

Fig. S3. Effect of elevated CO_2 on growth rate of *Pseudomonas syringae* pv. *tomato* DC3000 (*Pst*DC3000). Bacterial cultures were grown in LB broth without antibiotics for 32 h and OD_{600} was measured every four hours using a portable spectrophotometer. Two experimental replicates were conducted simultaneously, for each CO_2 concentration, in independent CO_2 chambers. Data points represent the average OD_{600} (optical density at 600 nm) between the two chambers. P-value was computed based on an F-test for the interaction of CO_2 and time in linear mixed effect model analysis using data after 4 hours due to unequal variance. The letter e denotes an exponent to the power of 10.

Fig. S4 Accumulation of salicylic acid (SA) and jasmonic acid (JA) at 6 h post-inoculation with *Pseudomonas syringae* pv. *glycinea* (*Psg*) in *a*CO_2 and *e*CO_2.

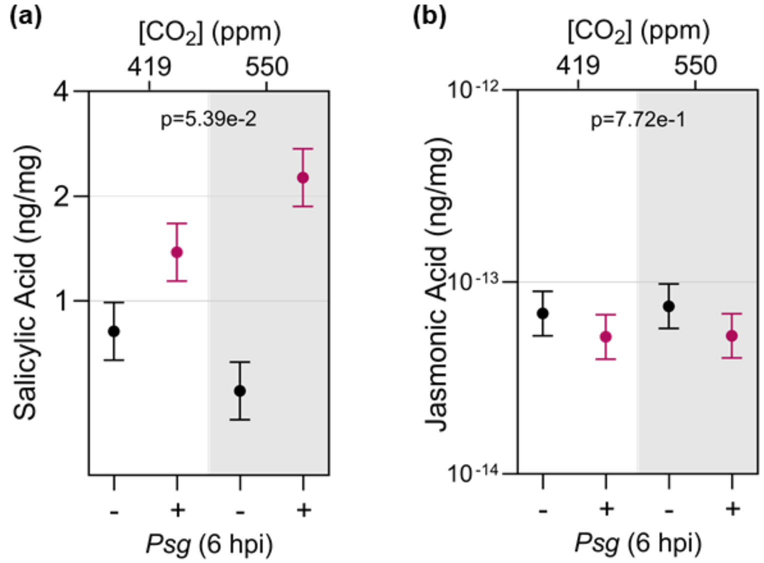

Fig. S4. Accumulation of salicylic acid (SA) and jasmonic acide (JA) at 6 h post-inoculation (hpi) with *Pseudomonas syringae* pv. *glycinea* (*Psg*) in *a*CO_2 and *e*CO_2. (a) SA and **(b)** JA levels were quantified using non-targeted and targeted metabolite analysis, respectively (Methods S1). Experiments were conducted independently, three times, using 8 plants per treatment for each CO_2 concentration. Data points represent the mean value across the three replicates and error bars represent the standard error. P-value was computed based on an F-test for the interaction of CO_2 and *Psg* treatment in linear mixed effect model analysis on log-transformed data. The letter e denotes an exponent to the power of 10.

Fig. S5 Hierarchical clustering of 21,822 DEGs (FDR < 0.01) responding to *Pseudomonas syringae* pv. *glycinea* (*Psg*) at 6 h post-inoculation in plants grown in *e*CO_2 (550 ppm) or *a*CO_2 (419 ppm) conditions.

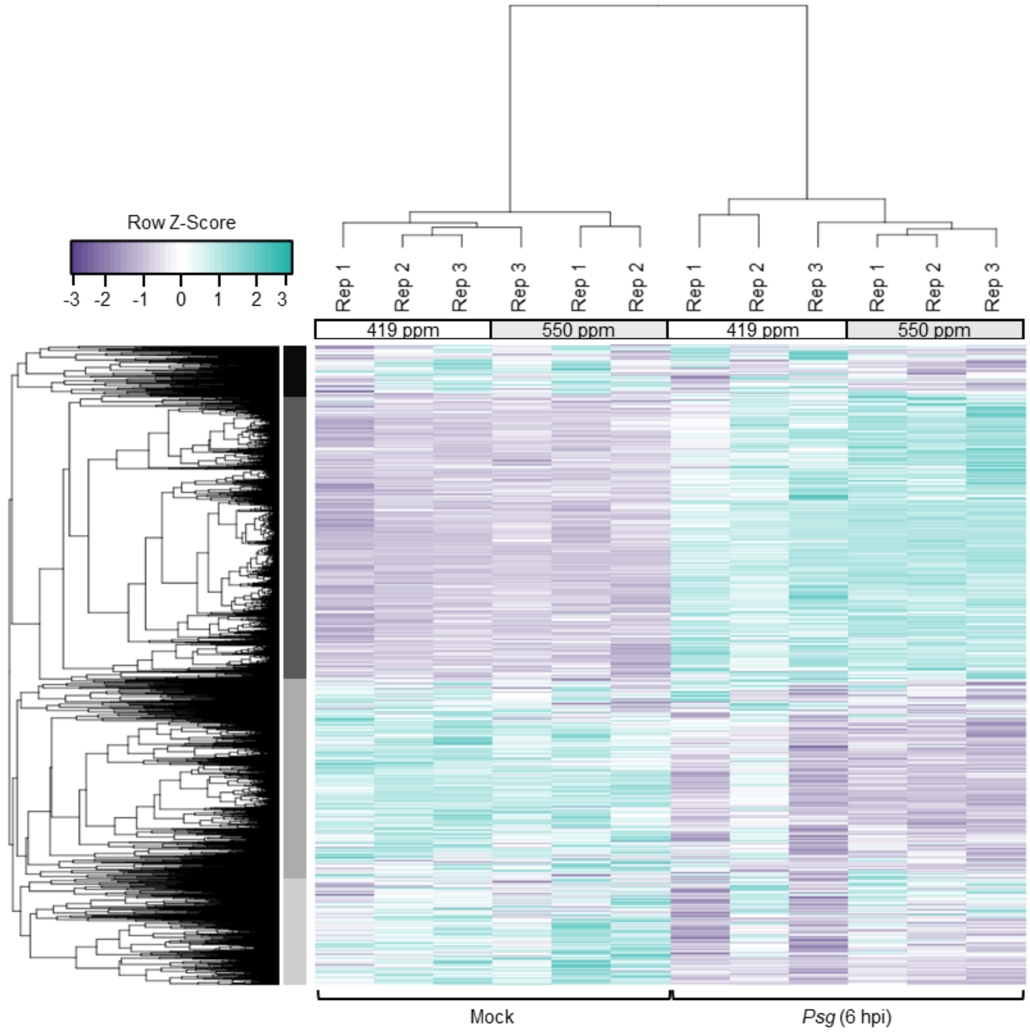

Fig. S5. Hierarchical clustering of 21,822 DEGs (FDR < 0.01) responding to *Pseudomonas syringae* pv. *glycinea* (*Psg*) at 6 h post-inoculation (hpi) in plants grown in *e*CO_2 (550 ppm) or *a*CO_2 (419 ppm) conditions. The full list of DEGs is provided in Table S7. Samples for 3' mRNA-Seq analysis were taken from the unifoliate leaves of 14-day old plants at 6-hpi with mock treatment or *Psg*. The three independent replicates were conducted using eight plants per CO_2 treatment. Rep indicates independent biological replicates. Row Z-scores were used for hierarchical clustering of DEGs, based on expression across samples and replicates. Purple indicates expression values below the row mean and teal indicates expression values above the row mean. Four different expression clusters were identified. These are indicated by differently shaded gray boxes to the left of the heat map.

Fig. S6 Effect of elevated CO_2 on the shoot fresh weight and shoot dry weight of soybean plants infected with bean pod mottle virus (BPMV) and soybean mosaic virus (SMV).

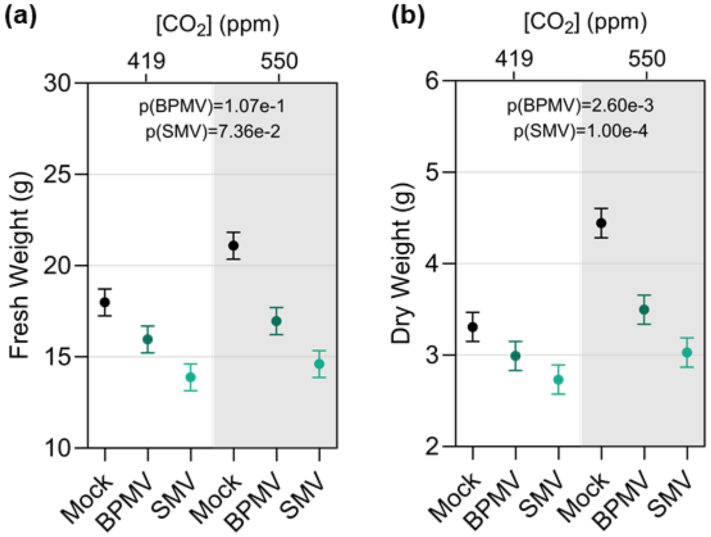

Fig. S6. Effect of elevated CO_2 on the shoot fresh weight and shoot dry weight of soybean plants infected with bean pod mottle virus (BPMV) and soybean mosaic virus (SMV). (a) Fresh weight and **(b)** dry weight of soybean plants at 21 days post-inoculation with BPMV, SMV, or mock treatment. Three experiments were conducted simultaneously in independent CO_2 control chambers, using 8 plants per treatment for each CO_2 concentration according to a replicated complete design. Data points represent the mean value across the three replicates and error bars represent the standard error. P-values were computed based on t-tests for comparing the difference between virus and mock under eCO_2 with that under *a*CO_2 in linear mixed effect model analysis. The letter e denotes an exponent to the power of 10.

Fig. S7 Bean pod mottle virus (BPMV) and soybean mosaic virus (SMV) accumulation at 21 days post-inoculation in soybean plants growing in *a*CO_2 and *e*CO_2.

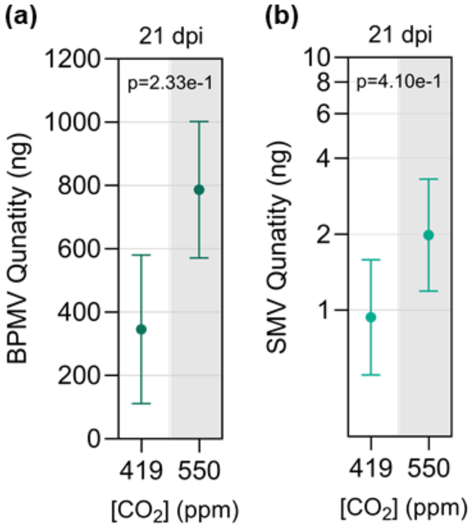

Fig. S7. Bean pod mottle virus (BPMV) and soybean mosaic virus (SMV) accumulation at 21 days post-inoculation (dpi) in soybean plants growing in *a*CO_2 and *e*CO_2. (a) BPMV and **(b)** SMV quantity was determined by RT-qPCR using multiplexed probes for BPMV or SMV and *Skp1* as an internal reference control. Samples were taken from the newest fullest expanded leaf. Three experiments were conducted simultaneously in independent CO_2 chambers, using 8 plants per infection treatment for each CO_2 concentration according to a replicated complete block design. Data points and bars represent mean group values and standard error across the three replicates. P-value for BPMV was computed based on an F-test for the main effect of CO_2 in linear mixed effect model analysis. P-value for SMV was computed based on an F-test for the main effect of CO_2 in linear mixed effect model analysis on log-transformed data. The letter e denotes an exponent to the power of 10.

Fig. S8 Effects of *e*CO_2 on root and shoot biomass of soybean plants infected with *Fusarium virguliforme*.

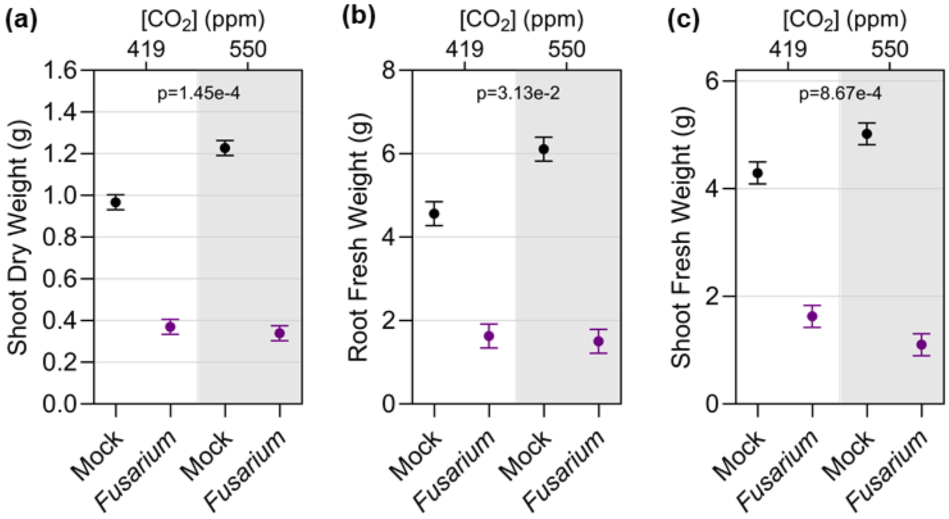

Fig. S8. Effects of *e*CO_2 on root and shoot biomass of soybean plants infected with *Fusarium virguliforme*. (a) Shoot dry weight, **(b)** root fresh weight and **(c)** shoot fresh weight at 35 days after germination in soil infested with *F. virguliforme* or mock infested. The three replicate experiments were conducted simultaneously in independent CO_2 control chambers using 6 plants per treatment for each CO_2 condition according to a replicated complete block design. Data points represent mean values with standard error across the three replicates. P-values were computed based on F-tests for the interaction effect between CO_2 and *Fusarium* treatment in linear mixed effect model analysis. The letter e denotes an exponent to the power of 10.

Fig. S9 Expression of the JA marker gene, *KTI1*, in roots of plants infected with *Fusarium virguliforme* in *a*CO_2 and *e*CO_2.

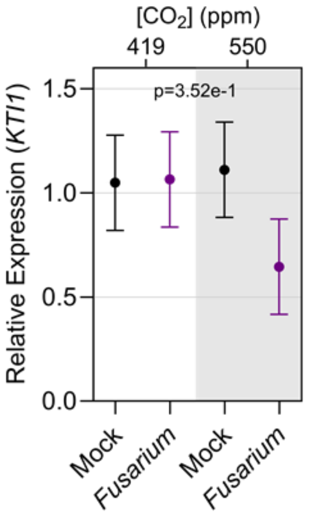

Fig. S9. Expression of the JA marker gene, *KTI1*, in roots of plants infected with *Fusarium virguliforme* in *a*CO_2 and *e*CO_2. RT-qPCR analysis was conducted on RNA extracted from root tissue using gene-specific primers for the JA marker gene *KTI1* with *Skp1* as an internal reference control. Experiments were conducted using 6 plants per treatment for each CO_2 concentration. Triplicate experiments were conducted simultaneously in independent CO_2 chambers. Data points and bars represent mean values and standard error across the three replicates. P-value was computed based on an F-test for the interaction effect between CO_2 and *Fusarium* treatment in linear mixed effect model analysis. The letter e denotes an exponent to the power of 10.

Fig. S10 CO_2 concentration does not impact *in vitro* growth of *Fusarium virguliforme*.

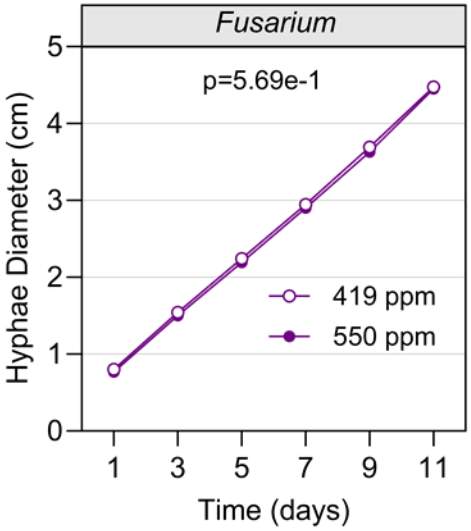

Fig. S10. CO_2 concentration does not impact *in vitro* growth of *Fusarium virguliforme*. Hyphae diameter was measured every 2 days over 11 days. Three experiments were conducted simultaneously using 10 plates per experiment, for each CO_2 treatment. Data points represent mean values across three replicates conducted simultaneously in independent CO_2 control chambers. P-value was computed based on an F-test for the interaction effect between CO_2 and time in linear mixed effect model analysis. The letter e denotes an exponent to the power of 10.

Fig. S11 Soybean root and shoot biomass is impacted by *e*CO_2 in *Pythium sylvaticum*-infected plants.

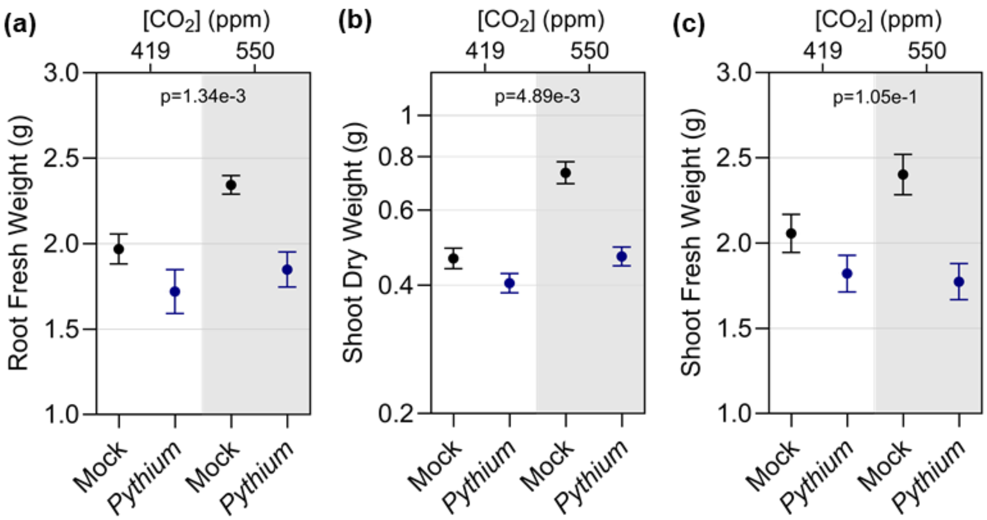

Fig. S11. Soybean root and shoot biomass is impacted by *e*CO_2 in *Pythium sylvaticum*-infected plants (a) Root fresh weight, **(b)** shoot dry weight and **(c)** shoot fresh weight in mock or *P. sylvaticum*-infected plants 21 days after inoculation. Experiments were conducted in triplicate using 6 plants per treatment, for each CO_2 condition. Triplicate experiments were conducted simultaneously in independent CO_2 controlled chambers. Data points represent mean values across the three replicates with standard error bars. P-values were computed based on F-tests for the interaction effect between CO_2 and *Pythium* treatment in linear mixed effect model analysis. Fourth power and log transformation were applied for root fresh weight and shoot dry weight respectively due to unequal variance on the original scale. The letter e denotes an exponent to the power of 10.

Fig. S12 *In vitro* growth of *Pythium sylvaticum* is accelerated under *e*CO_2.

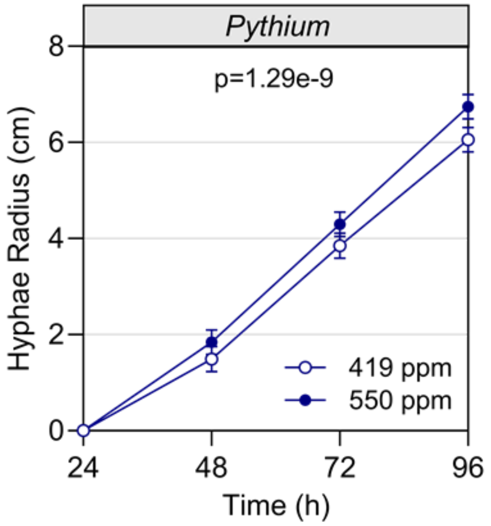

Fig. S12. *In vitro* growth of *Pythium sylvaticum* is accelerated under *e*CO_2. Experiments were conducted using 10 plates per treatment for each CO_2 condition in triplicate. The three experiments were done simultaneously in independent CO_2 controlled chambers. Hyphae radius was measured every day over 4 days. Data points and bars represent mean values and standard error across the three replicates. P-value was computed based on an F-test for the interaction effect between CO_2 and time in linear mixed effect model analysis. The letter e denotes an exponent to the power of 10.

Fig. S13 Expression of the SA marker gene, *PR1*, in roots of plants infected with *Pythium sylvaticum* in *a*CO_2 and *e*CO_2.

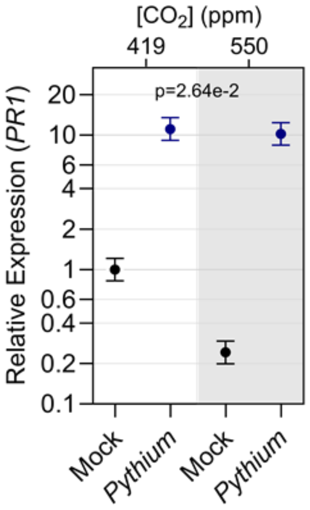

Fig. S13. Expression of the SA marker gene, *PR1*, in roots of plants infected with *Pythium sylvaticum* in *a*CO_2 and *e*CO_2. RT-qPCR analysis was conducted using RNA extracted from soybean roots 14 days after inoculation using gene specific primers for the SA marker gene *PR1* and *Skp1* was an internal reference control. Experiments were conducted simultaneously in three independent CO_2 controlled chambers using 6 plants per treatment for each CO_2 concentration according to a replicated complete block design. Data points represent mean values across the three replicates with standard error. P-value was computed based on an F-test for the interaction effect between CO_2 and *Pythium* treatment in linear mixed effect model analysis on log-transformed data. The letter e denotes an exponent to the power of 10.

Table S1 List of primers and probes used in this study.

**Table S1.** List of primers and probes used in this study.		
**Name**	**Oligonucleotide primer and Taqman probe (5'-3') sequences**	**Citation**
BPMV	F: GAGAGTTGCTGCTTGGAAGA	This study
	R: GTACCAATGAGGCCCAATCA	
	P: FAM/TGTGATGTC/ZEN/TGGGAATGCAGCAGT/IABkFQ[a]	
SMV	F: GAGAAGTTTACCCGATGGATGG	This study
	R: CATTCAGTAGTAAGGGCGTCAG	
	P: FAM/ACTGTCATG/ZEN/CAGATGGCTCACAGT/IABkFQ[a]	
GmPR1	F: GCTATGCTAATCAACGCAAAGG	This study
	R: ACTGCATCTGTGCCACTTAG	
	P: HEX/ACTGCCAAC/ZEN/TGATCCACTCTGGTG/IABkFQ[b]	
GmKTI	F: CCCATATCGAGTTCCTGTTATCC	This study
	R: GTCCTTCTGGTTGGCCATTA	
	P: TexRd-XN/CCCTTCGTTCTGAGTGGACCGT/IAbRQSp[c]	
GmSkp1	F: GAGCTCTCAGCACTAGGAATG	This study
	R: CAGGTCTGCCTCTAGAATCATC	
	P: Cy5/TCAGCAACAGTAACCACTGTAGCAATAGC/IAbRQSp[d]	
Pythium sylvaticum	F: GAAGGTGAAGTCGTAACAAGG	(Cooke et al., 2000)
	R: AGCGTTCTTCATCGATGTGC	
Fusarium virguliforme	F: GTAAGTGAGATTTAGTCTAGGGTAGGTGAC	(Wang et al., 2015)
	R: GGGACCACCTACCCTACACCTACT	
GmDCL2	F: GTTGCCCTCTACAGCCTATTT	This study
	R: CTTTAGTGACACGCTACCTCTG	
GmAGO1	F: CAAGATCCAGTCAGAGGAACAG	This study
	R: GCAGCAAGGTGAGCATAGTA	

[a]FAM = 6-Carboxyfluorescein, IABkFQ = ZEN-Iowa Black FQ
[b]HEX = Hexachlorofluorescein, IABkFQ = ZEN-Iowa Black FQ
[c]TexRd-XN = Texas Red-X NHS ester, IAbRQSp = Iowa Black RQ-Sp
[d]Cy5 = Cyanine-5, IAbRQSp = Iowa Black RQ-Sp

Table S2 SDS rating scale of foliar symptoms ranging from 0 to 7.

**Table S2.** SDS rating scale of foliar symptoms ranging from 0 to 7 (Roth et al., 2019).	
**Score**	**Foliar symptoms**
0	No visible symptom
1	1 to 10% of leaf surface chlorotic and/or 1 to 5% necrotic
2	10 to 20% chlorotic and/or 6 to 10% necrotic
3	20 to 40% chlorotic and/or 11 to 20% necrotic
4	40 to 60% chlorotic and/or 21 to 40% necrotic
5	> 60% chlorotic and/or > 40% necrotic
6	premature leaf drop up to one-third defoliation
7	premature leaf drop up to two-thirds defoliation

Table S3 Disease rating scale of *P. sylvaticum* root symptoms.

**Table S3.** Disease rating scale of *P. sylvaticum* root symptoms (Zhang & Yang, 2000)	
**Score**	**Root symptoms**
0	roots were long and full with no signs of discoloration or infection by the pathogen
1	roots were slightly stunted and moderately discolored
2	roots were severely stunted and may be severely discolored
3	roots were very short and rotted
4	seed was rotted and had not germinated

Methods S1 QuantSeq 3'mRNA-Seq data analysis and jasmonic acid (JA) and salicylic acid (SA) quantification.

Bioinformatic and Statistical Analysis of 3' mRNA-Seq data

For each of the 3' mRNA-Seq datasets (48 and 96 libraries), libraries were each sequenced on two lanes, generating two sequence files per library. Files were inspected with FASTQC (de Sena Brandine & Smith, 2019) to confirm sequence quality and quantity. Trimmomatic was used to remove adaptor sequences, low quality bases and reads less than 50 bp in length (Bolger *et al.*, 2014). STAR version 2.7.10 (Dobin *et al.*, 2013) was used to align reads to the Williams 82 reference genome sequence (Wm82.a4, (Schmutz *et al.*, 2010)). Samtools version 1.17 (Li *et al.*, 2009) was used to merge read data from separate lanes and identify uniquely mapping reads within the combined mapping files (bam files). Mapping files were imported into RStudio version 4.2.3 (Dobin *et al.*, 2013) using Rsamtools (Bandyopadhyay & Thilmony, 2021). The gene feature file (GFF) corresponding to the reference genome was imported using rtracklayer (Lawrence *et al.*, 2009). The number of reads per sample aligning to each gene was counted using summarizeOverlaps and a count table for all expressed genes within a tissue was generated. Over 329 million and 659 million uniquely mapping reads were identified across QuantSeq datasets 1 and 2, respectively. Data were normalized using the Trimmed Mean of M values (Gupta *et al.*, 2021) in the Bioconductor package edgeR (Robinson & Smyth, 2007; Robinson *et al.*, 2010; McCarthy *et al.*, 2012; Zhou *et al.*, 2014). Only genes with log2 counts per million (cpm) > 1 in at least three replicates were used in the analysis. ggplot2 (Wickham, 2009) was used to generate principal components, biological coefficients of variance, and MA plots to visually compare sample replicates and ensure reproducibility (Dziuda, 2010). Two and eight libraries with less than 1 million uniquely mapping reads were removed from subsequent analyses from QuantSeq datasets 1 and 2, respectively, leaving a minimum of six plant replicates per condition. Following renormalization, edgeR was used to analyze differentially expressed genes (DEGs) among the different datasets. For QuantSeq dataset 1, we identified DEGs whose expression changed in response to CO_2 levels (*e*CO_2 levels/*a*CO_2) at 21 days while the false discovery rate (FDR) was controlled at 0.01. For QuantSeq dataset 2, we identified DEGs responding to *Psg* treatment

(*Psg*/mock) in control and *e*CO_2 conditions and DEGs whose expression changed in response to CO_2 in *Psg*-treated (*Psg e*CO_2/*Psg* control CO_2) or mock-treated samples (Mock *e*CO_2/*a*CO_2) while controlling FDR at 0.01. Hierarchical clustering using hclust (Murtagh, 1985) was used to group similarly expressed genes from DEG lists of interest. Heatmaps were generated using Heatmap.2 in ggplot2 (Wickham, 2009).

DEG Annotation and Analysis of Overrepresented Transcription Factors and Promoters

DEGs were annotated using the SoyBase Gene Annotation Lookup Tool for Wm82.a4 (https://www.soybase.org/genomeannotation/). For given DEGs of interest, the best BLASTP Arabidopsis hits (TAIR version 10) from the annotation were used to query the STRING database (Szklarczyk *et al.*, 2023). To identify transcription factors (TFs) within DEG lists of interest, we used custom perl scripts to query *G. max* transcription factor information downloaded from the Plant Transcriptional Regulatory Map/Plant Transcription Factor Database (PlantRegMap/PlantTFDB v5.0, (Tian *et al.*, 2020), https://plantregmap.gao-lab.org). To identify TF binding sites significantly overrepresented within promoters of DEGs of interest we used the PlantRegMap TF enrichment tool (https://plantregmap.gao-lab.org/tf_enrichment.php) for *G. max*, using all available methods and threshold p-value < 0.05.

Single Phase Extraction for Metabolite Analyses of JA and SA

Approximately 100 mg of each sample was ground and spiked with an internal standard containing 10 µg nonadecanoic acid (1 mg/mL in ethanol) and JA-d5 (Cayman Chemical, Ann Arbor, MI) (JA-d5) (2.5 µg/mL in ethyl acetate). The extraction was initiated with the addition of 90% ice cold methanol using a modified version of the methanolic extraction protocol established previously (A *et al.*, 2005).

GC-MS Acquisition and Analysis for SA

Six hundred microliters of the combined extracts were aliquoted and dried using a speed-vac concentrator for 10 hours and then derivatized according to established protocols (Koek *et al.*, 2006). Gas Chromatography Mass Spectrometry (GC-MS) analysis was performed with an Agilent

6890 gas chromatograph coupled to a model 5973 Mass Selective Detector (Agilent Technologies, Santa Clara, CA) using a HP-5MSI 5% phenyl methyl silox with 30 m × 250 µM × 0.25 µm film thickness (Agilent Technologies). Identification and quantification were conducted using AMDIS (Automated Mass spectral Deconvolution and Identification System, National Institute of Standards and Technology, Gaithersburg, MD) with a manually curated, retention indexed GC-MS library with additional identification performed using the NIST20 and Wiley 11 GC-MS spectral library (Agilent Technologies, Santa Clara, CA). Final quantification was calculated by integrating the corresponding peak areas relative to the area of the nonadecanoic acid internal standard. Raw data were normalized to the amount of tissue used.

LC-MS Acquisition for Targeted JA Analysis

An Oasis Hydrophilic-Lipophilic-Balanced (HLB) column (30 mg sorbent) (Waters Corporation, Milford, MA) was used for the purification of JA as established previously (Kojima *et al.*, 2009; Kojima & Sakakibara, 2012). Liquid chromatography (LC) separations were performed with an Agilent Technologies 1290 Infinity II UHPLC instrument equipped with an Agilent Technologies ZORBAX Eclipse C18 (1.8 µm 2.1 mm × 100 mm) analytical column that was coupled to a 6470 triple quadrupole mass spectrometer with an electrospray ionization source (Agilent Technologies, Santa Clara, CA). A volume of 20 µL of each sample was injected into the LC system with the mass spectrometer in negative mode. Multiple reaction monitoring (MRM) was used for detection with transitions of m/z 209→59 (JA) and 214→62 (JA-d5), with fragmentation conducted at 25 eV for both JA and JA-d5.

References

A J, Trygg J, Gullberg J, Johansson AI, Jonsson P, Antti H, Marklund SL, Moritz T. 2005. Extraction and GC/MS analysis of the human blood plasma metabolome. *Analytical Chemistry* **77**: 8086–8094.

Bandyopadhyay A, Thilmony R. 2021. *Rice Genome Engineering and Gene Editing: Methods and Protocols*. 1st Ed.

Bolger AM, Lohse M, Usadel B. 2014. Trimmomatic: a flexible trimmer for Illumina sequence data. *Bioinformatics* **30: 2114–2120.**

Cooke DE, Drenth A, Duncan JM, Wagels G, Brasier CM. 2000. A molecular phylogeny of Phytophthora and related oomycetes. *Fungal Genetics and Biology* **30:** 17-32.

de Sena Brandine G, Smith AD. 2019. Falco: high-speed FastQC emulation for quality control of sequencing data. *F1000Research* **8**: 1874.

Dobin A, Davis CA, Schlesinger F, Drenkow J, Zaleski C, Jha S, Batut P, Chaisson M, Gingeras TR. 2013. STAR: ultrafast universal RNA-seq aligner. *Bioinformatics* **29**: 15–21.

Dziuda DM. 2010. *Data Mining for Genomics and Proteomics: Analysis of Gene and Protein Expression Data*. John Wiley & Sons.

Gupta K, Lalit M, Biswas A, Sanada CD, Greene C, Hukari K, Maulik U, Bandyopadhyay S, Ramalingam N, Ahuja G, *et al.* 2021. Modeling expression ranks for noise-tolerant differential expression analysis of scRNA-seq data. *Genome Research* **31**: 689–697.

Koek MM, Muilwijk B, van der Werf MJ, Hankemeier T. 2006. Microbial metabolomics with gas chromatography/mass spectrometry. *Analytical Chemistry* **78**: 1272–1281.

Kojima M, Kamada-Nobusada T, Komatsu H, Takei K, Kuroha T, Mizutani M, Ashikari M, Ueguchi-Tanaka M, Matsuoka M, Suzuki K, *et al.* 2009. Highly sensitive and high-throughput analysis of plant hormones using MS-probe modification and liquid chromatography-tandem mass spectrometry: an application for hormone profiling in *Oryza sativa*. *Plant & Cell Physiology* **50**: 1201–1214.

Kojima M, Sakakibara H. 2012. Highly sensitive high-throughput profiling of six phytohormones using MS-probe modification and liquid chromatography-tandem mass spectrometry. *Methods in Molecular Biology* **918**: 151–164.

Lawrence M, Gentleman R, Carey V. 2009. rtracklayer: an R package for interfacing with genome browsers. *Bioinformatics* **25**: 1841-2.

Li H, Handsaker B, Wysoker A, Fennell T, Ruan J, Homer N, Marth G, Abecasis G, Durbin R, 1000 Genome Project Data Processing Subgroup. **2009**. The Sequence Alignment/Map format and SAMtools. *Bioinformatics* **25**: 2078–2079.

McCarthy DJ, Chen Y, Smyth GK. **2012**. Differential expression analysis of multifactor RNA-Seq experiments with respect to biological variation. *Nucleic Acids Research* **40**: 4288–4297.

Murtagh F. **1985**. *Multidimensional Clustering Algorithms*. Springer.

Robinson MD, McCarthy DJ, Smyth GK. **2010**. edgeR: a Bioconductor package for differential expression analysis of digital gene expression data. *Bioinformatics* **26**: 139–140.

Robinson MD, Smyth GK. **2007**. Moderated statistical tests for assessing differences in tag abundance. *Bioinformatics* **23**: 2881–2887.

Roth MG, Noel ZA, Wang J, Warner F, Byrne AM, Chilvers MI. **2019**. Predicting Soybean Yield and Sudden Death Syndrome Development Using At-Planting Risk Factors. *Phytopathology* **109**: 1710–1719.

Schmutz J, Cannon SB, Schlueter J, Ma J, Mitros T, Nelson W, Hyten DL, Song Q, Thelen JJ, Cheng J, *et al.* 2010. Genome sequence of the palaeopolyploid soybean. *Nature* **463**: 178–183.

Szklarczyk D, Kirsch R, Koutrouli M, Nastou K, Mehryary F, Hachilif R, Gable AL, Fang T, Doncheva NT, Pyysalo S, *et al.* 2023. The STRING database in 2023: protein-protein association networks and functional enrichment analyses for any sequenced genome of interest. *Nucleic Acids Research* **51**: D638-D646.

Tian F, Yang D-C, Meng Y-Q, Jin J, Gao G. **2020**. PlantRegMap: charting functional regulatory maps in plants. *Nucleic Acids Research* **48**: D1104–D1113.

Wang J, Jacobs JL, Byrne JM, Chilvers MI. 2015. Improved Diagnoses and Quantification of *Fusarium virguliforme*, Causal Agent of Soybean Sudden Death Syndrome. *Phytopathology* **105:** 378-387.

Wickham H. **2009**. *ggplot2: Elegant Graphics for Data Analysis*. Springer Science & Business Media.

Zhang BQ, Yang XB. **2000**. Pathogenicity of Pythium Populations from Corn-Soybean Rotation Fields. *Plant Disease* **84**: 94–99.

Zhou X, Lindsay H, Robinson MD. **2014**. Robustly detecting differential expression in RNA sequencing data using observation weights. *Nucleic Acids Research* **42**: e91.
